# Co-existence of multiple trade-off currencies shapes evolutionary outcomes

**DOI:** 10.1371/journal.pone.0189124

**Published:** 2017-12-07

**Authors:** Alan A. Cohen, Caroline Isaksson, Roberto Salguero-Gómez

**Affiliations:** 1 PRIMUS Research Group, Department of Family Medicine, University of Sherbrooke, Sherbrooke, Québec, Canada; 2 Evolutionary Ecology unit, Department of Biology, Lund University, Lund, Sweden; 3 Department of Animal & Plant Sciences, University of Sheffield, Alfred Denny Building, Sheffield, United Kingdom; 4 School of Biological Sciences, Centre for Biodiversity and Conservation Science, The University of Queensland, St Lucia, Queensland, Australia; University of Thessaly School of Agricultural Sciences, GREECE

## Abstract

Evolutionary studies often assume that energy is the primary resource (*i*.*e*. “currency”) at the heart of the survival-reproduction trade-off, despite recent evidence to the contrary. The evolutionary consequences of having a single trade-off currency *versus* multiple competing currencies are unknown. Using simulations, we modeled the evolution of either a single physiological currency between reproduction and survival, or of multiple such currencies. For a wide array of model specifications varying functional forms and strengths of the trade-offs, we show that the presence of multiple currencies (e.g. nutrients, time) generally results in the evolution of higher lifetime reproductive success through partial circumvention of such trade-offs. Evolution of the underlying physiology is also more highly contingent with multiple currencies. These results challenge the paradigm of a single survival-reproduction trade-off as central to life history evolution, suggesting greater roles for physiological constraints and contingency, and implying potential selection for evolution of multiple trade-off currencies.

## Introduction

Life history trade-offs—compromises in the allocation of limited resources toward fitness components such as survival or reproduction—lie at the foundations of ecological and evolutionary research [1]. The physiological mechanisms that underlie such life history trade-offs have long been a subject of interest [2–4]. In particular, the evolution of aging is generally understood as a product of survival-reproduction trade-offs [5], and the physiological mechanisms underlying aging are thus thought to be related to the physiological mechanisms underlying this trade-off. Energy has long been treated as the primary limiting resource for this trade-off [6–8]. Indeed, many proposed aging mechanisms (oxidative stress, insulin signalling, mitochondrial dysfunction) are related to energy metabolism [5]. However, the potential limiting role of multiple nutrients has long been recognized in plant ecology [9–11]. In animals as well, many micronutrients are precursors for different vital biomolecules such as the amino acid cysteine, α-linoleic acids, and carotenoids, apparently independent of energy, and these may mediate trade-offs, such as carotenoid-dependent sexually-selected displays *versus* immunity [12]. The geometric framework for nutritional ecology has also established a clear basis for understanding nutrient composition in a multivariate nutrient space rather than as a simple function of calories [13], and joint responses to multiple nutrient inputs are increasingly thought to underlie life history variation [14]. Here we assess the impacts of trading off multiple resources simultaneously, both for aging specifically and for our understanding of trade-offs more generally.

**Fig 1:**
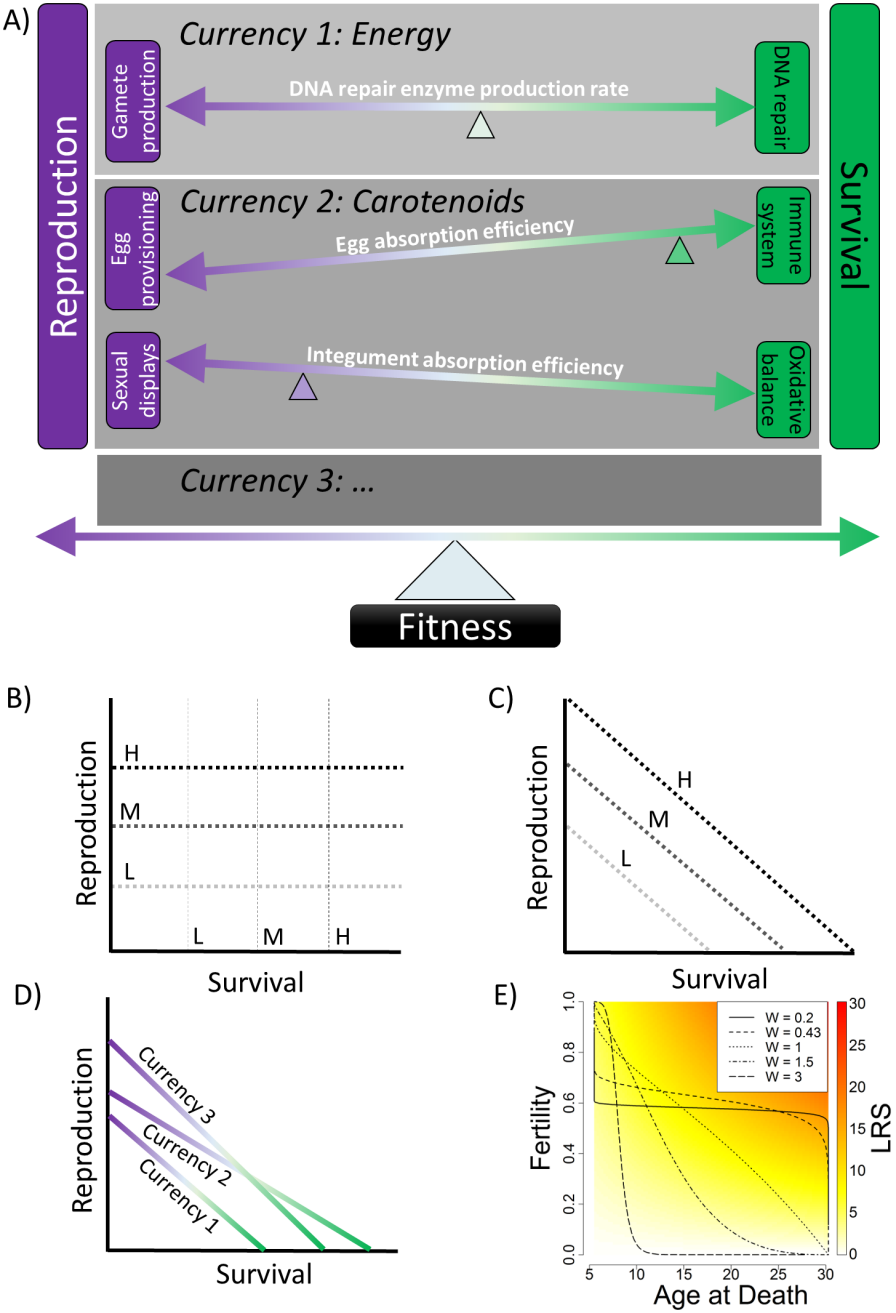
Conceptual framework for the physiological principles underlying trade-offs. (A) All trade-offs exist in the context of an **optimized trait**, usually fitness. This trait is modulated by balancing two or more **component traits** such as reproduction and survival. The genetic control of the trade-off is achieved based on allelic variation in multiple physiological traits such as DNA repair enzyme production rates. For instance, in currency 1 (energy), the rate of DNA repair enzyme production is presumably under tight genetic control with allelic variation. Greater production and use of these enzymes should increase ATP consumption, leaving less available for other tasks including gamete production. These **physiological traits** can be grouped based on the limiting factor, or “**currency**,” that they rely on (e.g. energy, carotenoids, gray boxes). Within these groups, a single net impact on reproduction and survival can be calculated because of the shared currency. Each **physiological trait** takes on a **value** (*PTV*) in each individual, assumed here to be fixed across an individual’s life. This *PTV* indicates how much the physiological trait favours reproduction *vs*. survival, as depicted by the slopes of the lines. A crucial **weight** parameter (fulcra, as positioned left-to-right), indicating how much reproduction could be gained for each unit loss in survival and *vice versa*. (B-C) Not all limiting factors are currencies, and limiting factors can (but do not always) vary in abundance (High: black, Medium: dark gray, Low: light gray lines). In (B), one limiting factor affects only reproduction (dotted lines) while the other affects only survival (dashed lines), showing that not all limiting factors need be currencies. In (C), the limiting factor is a currency trading off between reproduction and survival as indicated by the negative slope of the clines; any variation in abundance of the resource could cause positive co-variance between the fitness components [8]. Note, however, that not all currencies vary in abundance. Time, for example, is generally available in a fixed quantity equal across individuals. (D) Clines in (B) and (C) indicate different abundances of a single currency; clines in (D) indicate different currencies. Currencies vary in how constraining they are, with currencies #2 and #3 are less constraining than currency #1. Different currencies can also have different weights (*W* in our model, Eqs 3–6), as indicated by their slopes. Currency #3 is overall as constraining as currency #2, but its weight favours reproduction over survival, as shown by its steeper slope. In our simulations, variation in a *PTV* parameter across individuals is variation along a single cline, representing genetic variation in how the currency is invested in survival *vs*. reproduction. *W*s are fixed within a given simulation (constant slope of the cline), and constant resource availability is assumed (constant intercept of the cline). *W* is thus constant for the population, whereas *PTV* varies across individuals. (E) More realistic models of currency clines are non-linear but decline monotonically. This panel shows the relationship between fertility and expected age at death for actual curves of *PTV*s for the values of *W* as used in our models across *PTV* from -10 (bottom right) to +10 (top left). Background shading indicates the expected lifetime reproductive success **(***LRS)* based on the fertility and age at death. Selection is expected to adjust each *PTV* along its curve to the “reddest” point (highest fitness). Not all clines pass through equally high maximum fitness points. As a result, lower values of *W* are generally favoured by selection (see Results).

To explore these questions, we first construct a theoretical framework (Fig 1A) to link trade-offs to underlying physiological principles. Although our framework can be applied to any trade-off, we develop it specifically in the context of the survival-reproduction trade-off thought to underlie aging [5, 15]. The starting point is an optimized trait (usually fitness) that requires balancing two or more component traits that are traded off (e.g. survival *vs*. reproduction, or growth *vs*. maintenance *vs*. reproduction [16–18]). The trade-off arises because certain limiting factors can only be allocated to one component trait at the expense of the other(s) [19]; we call these limiting factors “currencies.” Note that not all limiting factors are currencies. For example, some factors might be limiting only for reproduction or survival (Fig 1B); only when there is a trade-off (Fig 1C) does the limiting factor become a currency. Likewise, some currencies may not vary in their availability. For example, time could be traded off between predator avoidance and mate searching, but there is always a fixed amount of time. Likewise, even in an environment with *ad libitum* food, energy is often limiting because of the capacity of the gut to process it [15]. However, in other cases, a currency may vary in availability, such that both component traits can be increased as availability increases (Fig 1C), as in Van Noordwijk and de Jong [8].

This framework is in broad agreement with Dynamic Energy Budget (DEB) theory, a theoretical framework providing substantial mechanistic detail on how the acquisition of multiple nutrients can link processes and functions at levels from molecules to ecosystems [20, 21]. While the framework we present here is much less mechanistically detailed, it draws on similar physiological principles, and responds to a major question emerging from DEB theory: how does this mechanistic underpinning interact with evolutionary forces[21]? Notably, DEB theory explicitly incorporates the potential for trade-offs based on multiple currencies (nutrients) [20].

Here, we were interested in understanding how the number of currencies underlying a trade-off affects the way selection operates on the trade-off. We suppose that the key variation of interest is the physiological mechanisms allocating the currencies to different survival- or reproduction-related tasks, and that there is allelic variation in these mechanisms permitting selection to operate (Fig 1A). We distinguish this allelic variation in how currencies are allocated from environmental variation in the availability of resources/currencies. While undoubtedly interesting, this latter question exceeds the scope of this paper.

Each physiological trait can take different values (physiological trait values, PTVs) in different individuals. These PTVs indicate to what extent each trait favours reproduction or survival in each individual, represented schematically by the slopes of the lines in Fig 1A or by the position on the clines in Fig 1D. We suppose that each individual’s PTV for each trait is fixed for its lifespan. Lastly, we introduce a concept of “weights,” indicating how much reproduction could be gained for a unit loss in survival, or *vice-versa*. This concept is represented by the left-to-right positioning of the fulcra in Fig 1A, and by the slopes of the lines in Fig 1D. As a fulcrum moves right or a slope gets steeper, reproduction becomes “cheaper” relative to survival, meaning a small loss in survival can be accompanied by a large gain in reproduction. In reality, the clines in Fig 1D are likely non-linear, as shown by the functions describing the relationship between fertility and expected age at death in our model, for different weights (Fig 1E; see below).

As noted above, most theoretical consideration of trade-offs to date implicitly or explicitly assumes a single currency, usually energy (e.g. [8]), but empirically there is strong evidence for multiple currencies. We therefore wished to test whether the presence of multiple currencies might change our understanding of how trade-offs operate. Would multiple currencies reinforce or weaken the trade-offs? Would evolution of physiological traits become more or less deterministic? Using the general framework above, we have constructed a series of stochastic simulations to address such questions. We model individuals in a population over hundreds of generations, assuming genetic transmission of PTVs as the only vehicle for heritability of fitness components (survival and reproduction). We evaluate the evolution of fertility, lifespan, lifetime reproductive success (LRS), and the PTVs (Fig 1A).

Such an individual-level model is essential to incorporate appropriate stochasticity, as borne out by contingency in our results. The resulting model was inevitably complex (11 input parameters and 9 equations). A major challenge for such a model is the specification of realistic details: for example, are trade-offs linear, and if not, what functional form do they take? We address this challenge with a three-pronged strategy:

1. Reduce the number of mechanistic decisions by keeping the basic model as simple as possible to answer the core underlying question. For this reason, we ignore temporal variation in resource availability, genetic recombination through sexual reproduction, population dynamics such as demographic increases or declines, population structure, density dependence, or selective benefits of short generation time; we speculate about how our findings may be shaped by these factors in the *Discussion*. Some additional complexities have been tested in sensitivity analysis (age-dependent fertility, more than two currencies, *etc*.).
2. When possible, we incorporate existing theoretical or empirical knowledge. For example, there are certainly physiological constraints on how many offspring can be produced per unit time (a bird could not lay 1000 eggs per clutch). For this reason, we implement exponentially increasing costs to achieve extreme reproduction or survival values.
3. In the many cases where such knowledge is unavailable or uncertain, we test as diverse an array of parameterizations as possible (wide ranges of parameter values, Gompertz vs. Gompertz-Makeham vs. linear mortality models, *etc*.).

We carefully document the logic of all our choices and the results of a wide array of sensitivity analyses in the Supporting Information (SI). In the end, across thousands of model parameterizations (some not presented), we always found one of two possible outcomes: results qualitatively equivalent to those presented below, or model failure indicating unrealistic parameterisations (population crashes, evolution of nearly infinite reproductive rates, absence of a trade-off, *etc*.).

## Results

### Evolution of fitness components

**Fig 2:**
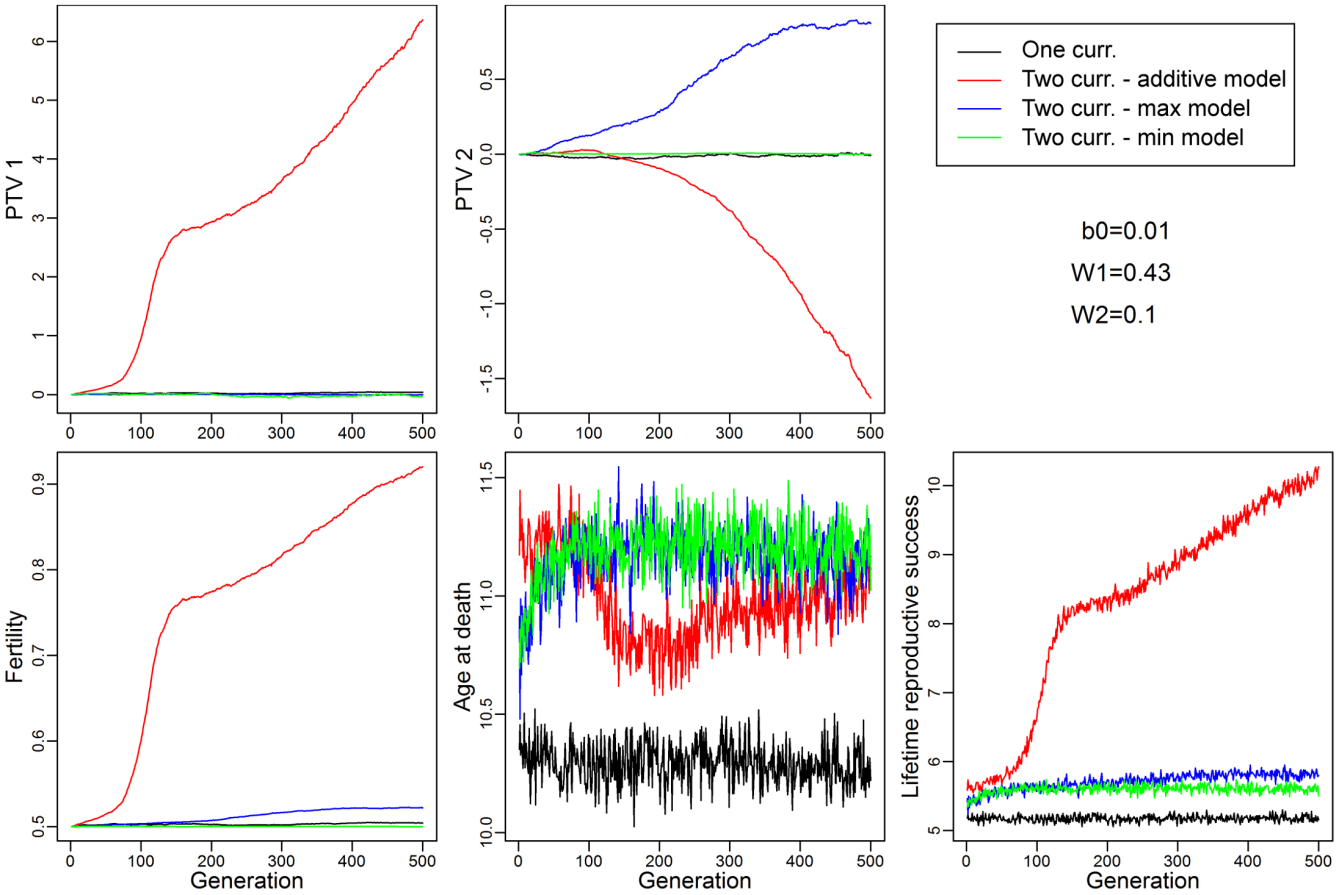
An example of a single run of our model to evaluate the effects of co-existing reproduction-survival trade-offs on the evolution of fertility, age at death and life time reproductive success. Fixing the trade-off weight *W*_*1*_ at 0.43 assures little evolution in *PTV*_*1*_ over the 500 generations; choosing a different value for *W*_*2*_ produces different outcomes in the two-currency scenarios. All parameters evolve differently under all four scenarios, implying important evolutionary consequences for the number of currencies and the interactions among them under the assumptions of our model (see Discussion).

Our core model traced the evolution of the mean of five individual traits over 500 generations in a stable population of 10,000 individuals: values of *PTV*_*1*_ and *PTV*_*2*_, *A*_*d*_, *f*, and *LRS*. These traits were followed in four scenarios for each parameterisation: a single trade-off currency, or two trade-off currencies that could interact in one of three ways (Additive, Maximum, and Minimum; see The model). The key input parameters were the weights *W*_*1*_ and *W*_*2*_ for *PTV*_*1*_ and *PTV*_*2*_ respectively. These weights modulate the increase in survival gained for each unit decrease in fertility for each PTV (*i*.*e*., large weights result in greater lifespan gained per unit of fertility lost; S7 Text, Fig 1 and S1–S3 Figs). Using graphical representations (Figs 1B–1D and 2), we identified five values of *W* that represented a broad range of survival-fertility relationships and were used in subsequent analyses. *W* = 0.43 was of particular interest because it represents evolutionary equilibrium in a one-currency model (*i*.*e*., *PTV* does not evolve away from 0). For any given parameterisation, model results were largely replicable across 100 repeated simulations (e.g. S4 Fig).

**Fig 3:**
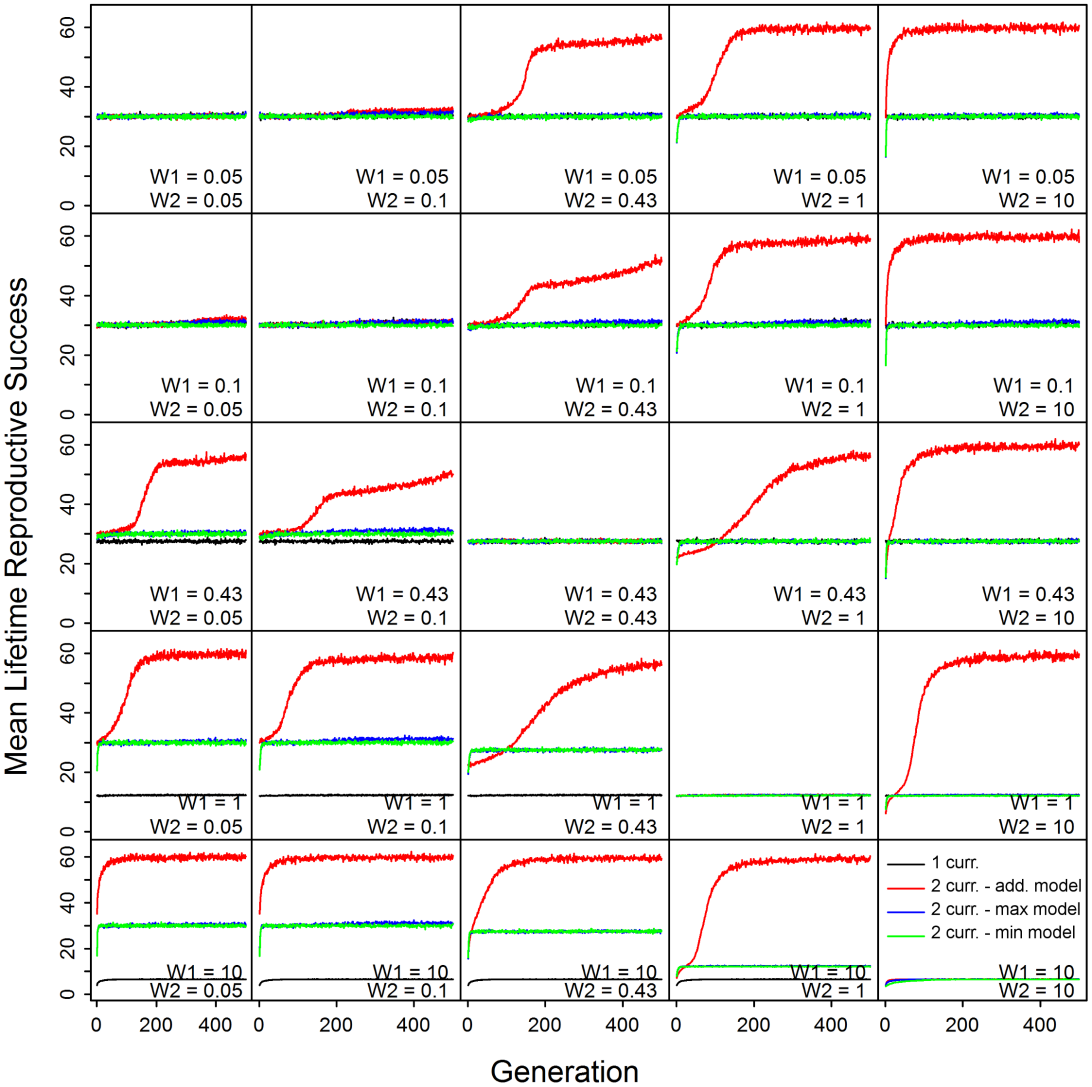
Changes in lifetime reproductive success (*LRS*) evolution across a variety of trade-off weight values (*W*_*1*_ and *W*_*2*_) for physiological trait values *PTV*_*1*_ and *PTV*_*2*_, with *b*_*0*_ fixed at 0.01, the minimum rate of ageing.

Across a wide range of models, we found that presence of two currencies resulted in the evolution of greater LRS than the single-currency model (e.g., Figs 2 and 3 and S5 Fig). Evolution of higher LRS in this context implies the trade-off is weaker (*i*.*e*., less constraining). This finding held for the Additive, Maximum, and Minimum two-currency models (see The model for details). The exceptions were when *W*_*1*_ = *W*_*2*_ (Additive model) or when *W*_*1*_ ≤ *W*_*2*_ (Maximum and Minimum models); in these cases, results were similar to the one-currency model.

### Evolution of physiological traits underlying trade-offs

**Fig 4:**
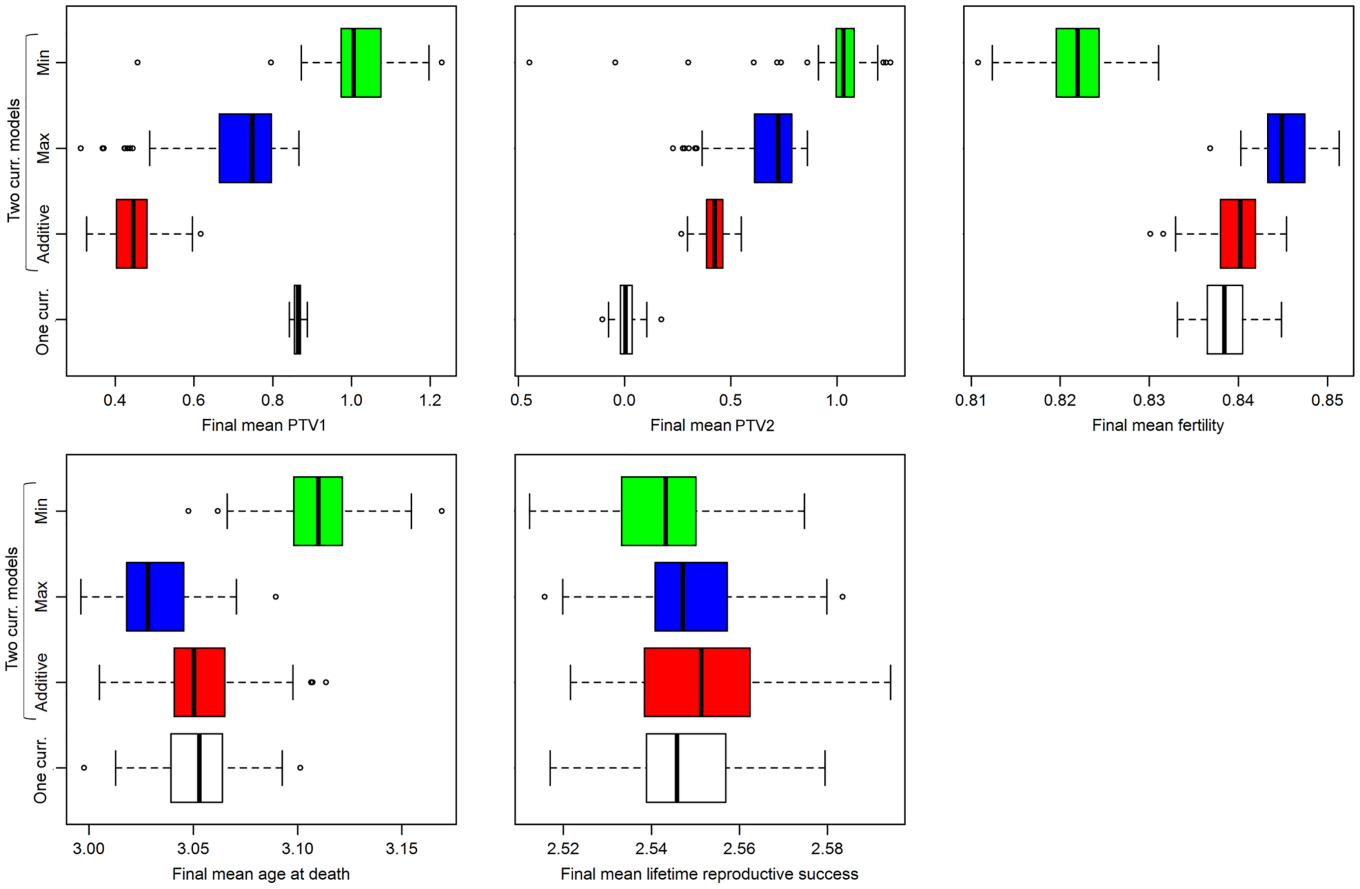
Variation of results across 100 identical simulations where *W*_*1*_ = *W*_*2*_ = 2. Because both trade-off weights are equal and far from the equilibrium value near 0.43, this parameterization allows us to examine how stochasticity and contingency might affect the physiological trait values when there should not be a tendency for one trait to evolve in a way systematically different from the other. As expected, results are similar across all four models for *LRS*. However, results are quite different for physiological trait values *PTV*_*1*_ and *PTV*_*2*_ across the four models, and there is also substantial stochastic variation within each model’s results across the 100 simulations. This confirms that both chance and the physiological details that determine trade-off functions can have major roles in determining how physiology evolves, even when life history traits are largely stable.

The underlying physiological traits *PTV*_*1*_ and *PTV*_*2*_ also showed markedly different evolutionary trajectories in single-currency and two-currency models (S6–S9 Figs). Unlike fitness, this result held even when *W*_*1*_ = *W*_*2*_. In order to explore this variation when both weights are far from the equilibrium value of 0.43, we ran 100 simulations where *W*_*1*_ = *W*_*2*_ = 2 (Fig 4). As expected, *LRS* after 500 generations differs little either across the four models or across the 100 runs. However, *PTV*_*1*_ and *PTV*_*2*_ do vary markedly both across models and runs. In particular, with a single currency, variance in *PTV*_*1*_ and *PTV*_*2*_ is minimal, but with two currencies the variance increases substantially. This implies that both physiological details (differences across models) and stochasticity/contingency (differences across runs) may be important factors determining the evolution of physiological traits even in the absence of clear selective pressures linked to fitness components. This variance in *PTV*_*1*_ and *PTV*_*2*_ in multiple-currency models was the sole exception to the replicability of our findings when running the same parameterisation multiple times.

### Model variations

**Fig 5:**
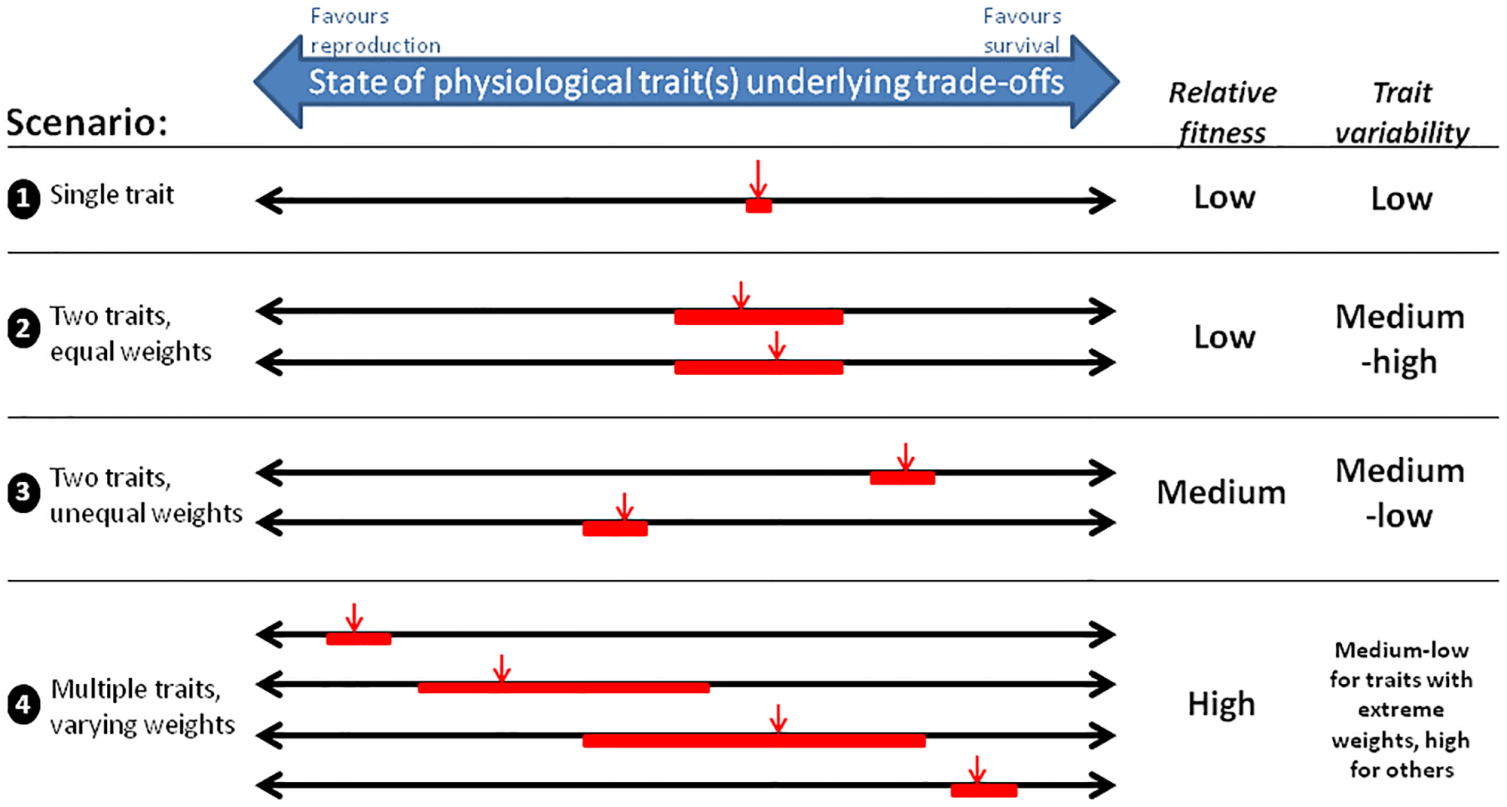
A summary of how multiple trade-off currencies affect evolutionary processes. This schematic presents a qualitative interpretation of our results based largely on the additive model. Under four trade-off scenarios, one or multiple lines represent currencies, the value of which (left to right; red arrow) represents the relative benefits for the fitness components reproduction and survival. Red bars represent potential values the trait might take at equilibrium/ evolutionary optimum. The position of each red bar on the line depends on the weight for that trait and on any other traits and their weights; when weights of multiple traits are equal, their bars are aligned. “Relative fitness” refers to expected fitness in the hypothetical scenario where, all else equal, individuals at evolutionary equilibrium from the four scenarios are mixed in a single population. “Trait variability” (red bars) refers to how large the role of chance and contingency is in determining final trait values. For example, a single trait has a single optimum and is under strong stabilizing selection (thus, a narrow bar). Two traits with equal weights can compensate for each other and can thus vary substantially for random reasons. When multiple traits are present, fitness is determined by the two most extreme values, which have much less variability than the others.

We ran several models where we extended the additive model to include three or four trade-off currencies (*i*.*e*., *PTV*_*3*_ and *PTV*_*4*_ with *W*_*3*_ and *W*_*4*_; S10 Fig). Generally, adding another currency had little effect on the overall model unless its weight was outside the range of the other currencies’ weights; results were thus determined largely by whichever two of the currencies had the largest and smallest weights (Fig 5). We also ran the original model incorporating a linear increase in fertility with age, but results were qualitatively equivalent to other models (S11 Fig).

## Discussion

Our simulations strongly and consistently confirm that the number of trade-off currencies present can have major impacts on how the trade-offs shape evolutionary processes. Specifically, compared to a single trade-off currency, the presence of multiple currencies diminished the force of the trade-offs and thereby allowed evolution of greater lifetime reproductive success. Simultaneously, multiple trade-offs rendered evolution of the underlying physiological traits more stochastic, with a much greater range of trait values possible across different runs of identical models. Given the clear empirical support for multiple trade-off currencies in nature [9, 10, 12, 14], these findings imply that trade-offs are likely to be substantially less important in structuring trait evolution than previously thought, leaving a larger role for physiological constraints. This is consistent with frequent failure to empirically detect expected trade-offs [22]. Likewise, contingency and chance are likely to have particularly large roles structuring the physiological traits underlying the trade-offs.

We developed the model around the specific example of survival-reproduction trade-offs thought to underlie aging. In this context, our findings imply that the evolution of lifespan should be at least partially decoupled from the evolution of reproductive investments. This is consistent with a growing consensus that, while there is a slow-fast continuum structuring life history evolution, there is also substantial variation around this axis and nuance in its structure [23]. Indeed, partial decoupling of allocations to survival and reproduction have been reported in many taxa: mammals [24, 25], birds [26], reptiles [27], ants (at the colony level) [28], and plants [18]. Our findings, though at the level of a single species, provide an explanation for this variation.

At a physiological level as well, our findings can help explain the complexity (even chaos) in the physiological ecology literature. There has been little success replicating links between physiological traits such as “immunocompetence” or oxidative balance and life history traits or fitness proxies, with findings often depending heavily on the species, the ecological context, or the biomarkers used [12, 29–33]; indeed, due to this complexity immunocompetence is no longer considered a valid concept [34, 35]. This is exactly as would be predicted in the presence of multiple trade-off currencies. Physiological evolution in response to selection for a given survival/reproduction balance should be both stochastic and contingent: the physiological variables should have multiple pathways through which they could achieve the balance. Expressed via the metaphor of fitness landscapes, a single trade-off currency creates a fitness peak, but multiple currencies create a higher ridge of equally fit trait combinations. This greatly complicates the task of understanding how physiology undergirds life history trade-offs.

Why would multiple currencies create such a ridge? Our core findings are summarized in Fig 5, and together provide an answer. The key is the weight parameter, which represents the relative benefits to survival and reproduction for a given change in a physiological trait. If by chance the two traits have identical weights, a given survival-reproduction balance could be achieved by a wide range of trait combinations (Fig 5 Scenario 2). If they don’t, the range of trait values may be constrained, but the overall fitness improves by using each physiological trait to obtain more of the fitness component that it delivers most efficiently. An intuitive analogy is a bank that offers Reward Points (RPs) which can be invested either in Savings or Spending. If you only have money (no RPs), there is a single optimum way to allocate it to Spending and Saving (Fig 5 Scenario 1). If you have both money and RPs, and if the amount of value in Spending and Savings that you can buy with RPs is equal (*i*.*e*., weights are equal), there are many ways you could allocate both the RPs and money to still arrive at your optimum allocation (Fig 5 Scenario 2). If you have both, but $ Savings / RP is not the same as $ Spending / RP (*i*.*e*., weights are not equal), you can take advantage of this to get a greater total value, using the RPs for their more advantageous allocation. However, variability of the optimal solution is again reduced (Fig 5 Scenario 3). If you were to get more types of RPs from different banks, the total value of your Savings and Spending would only depend on whichever two gave the most advantageous conversions at the two extremes (Fig 5 Scenario 4).

One of the most important implications of this study is that, even though trade-offs have historically been treated as a central tenet of ecology and evolution, they may in fact be much less constraining and straightforward than previously thought. Although our model shows the survival-reproduction trade-off, the conclusions should be valid for anytrade-off with multiple currencies. If the presence of multiple currencies allows organisms to circumvent the trade-offs, as is the case in all our models, physiological constraints on trait values may be much more important than trade-offs in limiting organismal evolution. This could be true even though gene knockdown experiments [36–39] or perturbation experiments, such as testosterone supplementation or increasing parental workload [40, 41], might show evidence for trade-offs [40, 41]. If each trade-off trait is optimised as part of an overall suite of interacting traits that together generate fitness components, it is not surprising that perturbing any single trait or knocking down a single gene would affect these fitness components and appear to support a trade-off model.

The model presented here ignores a number of key ecological and evolutionary processes, including variation in resource availability, sexual reproduction, genetics, density-dependence, structured population dynamics, eco-evolutionary dynamics, and overlapping generations. Nonetheless, the most important limitation to our model is less its eco-evolutionary simplicity than its physiological simplicity. As noted above, the different aspects of physiology underlying separate currencies are likely correlated (and thus constrained) for physiological reasons as well as by their effects on fitness components. Biologists are simply too far from being able to understand these constraints well enough to model them here.

We view the primary implications of our findings as theoretical: trade-offs are perhaps less important than once thought, while constraints are more important. This is reinforced by additional questions on resource limitation [8, 20], environmental contingency of trade-offs [42, 43], and the interactions among trade-offs at different hierarchical levels (e.g. physiological trade-offs and life history trade-offs), all of which together suggest that most real-world situations will involve a complex interplay between trade-offs, constraints, contingency, chance, and environment. Physiological evolution is also likely highly contingent and stochastic under certain conditions. Beyond these theoretical implications, there are also new directions for empirical research, particularly in experimental evolution (e.g. *Drosophila*, *Arabidopsis*, bacteria). Does imposition of a selection regime to favor survival or reproduction produce changes in pathways associated with multiple potential currencies (e.g. limiting nutrients)? Our results predict that these changes would impact different pathways differentially depending on how much survival vs. reproduction is optimized. Does *ad libitum* provision of a limiting nutrient reduce or strengthen a trade-off? We would predict that, in some cases, it should strengthen the trade-off after sufficient time for compensatory evolution. Lastly, we believe our results provide a caution to simplistic interpretation of experimental results. As noted above, experimental confirmation of a trade-off could actually arise even if the trade-off is relatively unimportant in evolutionary terms, due to the complex intersection of multiple currencies and multiple ecological processes.

## The model

### Overview

This section provides a broad conceptual overview of our model; formulas, details of our reasoning, and model assumptions are specified in subsequent sections and in the SI. Our model is based on a framework previously developed to understand how adaptation to urban stress might affect life history traits of organisms [44]. We used a stochastic model to simulate the evolution of individuals within a population with overlapping generations and a trade-off between their fertilities and survivals. Our time steps within generations can be thought of at any pertinent scale, but we refer to them as “years.” We fixed a number of individuals per generation (10,000) and generations (500) for each simulation (see S12 Fig for an example of 10,000 generations). Most parameterisations were run a single time after verifying the stability of results across 100 runs of several parameterisations (e.g. S4 Fig). We assumed that one or multiple trade-off currencies could decrease survival while increasing fertility, or *vice versa*. For the first generation, we generated random values of the physiological trade-off traits assumed to underlie the currency (*PTV*s hereafter; see S3 Text and S13 Fig). Currencies were weighted such that we could vary the increase in survival gained for each unit decrease in fertility (*i*.*e*., large weights mean that more lifespan can be gained for each unit of fertility lost; S7 Text, Fig 1, and S1–S3 Figs). We assume that effects on survival occur through aging rate (*i*.*e*., we ignore transient increases in mortality risk). Based on an individual’s *PTV*(s), we calculated aging rate to stochastically generate an age at death (S5 Text and S14 Fig), as well as fertility per unit time (constant throughout life, S6 Text). We assumed that physiological constraints impose limits on both lifespan and reproduction, implemented as asymptotic limits for the respective functions (S7 Text and S1 Fig). Lifetime reproductive success (*LRS* hereafter) is defined as the product of an individual’s fixed annual fertility and age at death, with a stochastic component (S6 Text and S15 Fig).

In order to maintain a stable population, each subsequent generation was created by sampling the *PTV*s of individuals in the previous generation weighted by the individual’s *LRS*, and with stochastic variation of the *PTV* based on a “heritability” parameter affecting fidelity of trait transmission across generations (S4 Text and S16 Fig). We kept track of the mean value of each key parameter (*i*.*e*., *PTV*(s), age at death, fertility, and *LRS*) for each generation (e.g. Fig 2). Currencies were modeled as either a single currency, or as two currencies that could interact with each other in one of three different ways (see below). All simulations were performed in R v. 3.0.0 and a fully commented script is provided in the SI.

### Calculation of age at death

Age at death for each individual was generated stochastically from a Gompertz probability distribution based on increasing mortality with age,

μ(x)=a×ebx (1)

where *μ(x)* is mortality at age *x*, *a* is initial mortality, and *b* is the rate of exponential increase in mortality. The aging rate (*b*) was determined by the trade-off functions (below). Sensitivity analyses with multiple other mortality functions and parameterisations, including a simple linear increase with age, consistently highlighted the robustness of our results (S5 Text and S17–S21 Figs).

### Calculation of fertility and lifetime reproductive success

Fertility per time unit was constrained to be non-negative and below a physiological constraint threshold, *f*_*max*_, fixed at 1 in all our models. Each individual’s fertility value (*i*.*e*., expected rate of reproduction per unit time) varied depending on the individual’s specific *PTV*(s). *LRS* for each individual was drawn from a normal distribution with the mean as the product of the individual’s age at death (*A*_*d*_) and fertility (*f*), and the standard deviation as 10% of this product (S6 Text and S15 Fig).

LRS~N(f×Ad,f×Ad×0.1) (2)

### Modelling trade-offs

We used four different models of currencies, one with a single currency and three with two currencies interacting in different ways. All four share a basic structure of how fertility and mortality are related: (1) An increase in the *PTV* must have a monotonically increasing association with both aging rate *b* and fertility *f*. (2) The relationship between the *PTV* and *b* should be exponential but not so sharp that we cannot detect meaningful variation in age at death across a range of *PTV*. This ensures that very high values of the *PTV* are associated with essentially instant death, giving an upper bound to reasonable values of the *PTV*. An exponential rather than linear increase is necessary to ensure diminishing marginal returns as fertility increases (otherwise infinite fertility can evolve). (3) There is a minimum value of *b* approached asymptotically as the *PTV* gets small, reflecting physiological constraints on the ability to avoid aging. This is both biologically realistic [45] and ensures model stability by avoiding the evolution of nearly infinite lifespan coupled with infinitesimal reproduction. (4) *f* should be a logistic (or other s-shaped) function of the *PTV*, bounded at the bottom by zero and at the top by *f*_*max*_. *f*_*max*_ reflects physiological constraints on reproductive rate, such as the number of eggs or seeds that can be generated per unit time.

Based on these principles, in the single currency model,

$$b\;=\;b_{0}+e^{\frac{PTV+\gamma }{W}}$$ (3)

and

$$f\;=\;\frac{f_{max}}{1\;+\;e^{-PTV∙W}}$$ (4)

where *b*_*0*_ is the minimum value of *b* (a physiological constraint on minimum aging rate), *f*_*max*_ is the maximum (asymptotic) value of fertility, *PTV* is a value of the physiological trait underlying the trade-off currency (higher favours reproduction, lower favours survival), *W* is the weight of the currency (fixed in each simulation), and *γ* is a fixed parameter that allowed us to align Eqs (3) and (4) so as to ensure a meaningful trade-off (see S7 Text, S1, S2, and S22 Figs). *W* is a key parameter because it determines how much fertility can be obtained for a unit of lifespan, and *vice versa*; low *W* favours slow pace of life, high *W* favours a live-fast, die-young strategy (Fig 1).

In the two-currency models, each individual has a *PTV* for each of two currencies, *PTV*_*1*_ and *PTV*_*2*_, both simulated for the first generation as above, *i*.*e*., drawn from the normal distribution *N*(0, 0.1). No co-evolutionary constraints are imposed on *PTV*_*1*_ and *PTV*_*2*_ other than through maximisation of fitness. Each currency has its own weight, *W*_*1*_ and *W*_*2*_, fixed in each run of the model. In the first two-currency model, the effects of the physiological trade-off traits are additive as described in Eqs (5) and (6).

$$b\;=\;b_{0}+e^{\left(\frac{{PTV}_{1}+\;\frac{\gamma }{2}}{W_{1}}\;+\;\frac{{PTV}_{2}+\;\frac{\gamma }{2}}{W_{2}}\right)}$$ (5)

$$f\;=\;\frac{f_{max}}{1\;+\;e^{\left({-PTV}_{1}∙W_{1}\;{-\;PTV}_{2}∙W_{2}\right)}}$$ (6)

Note that because of the exponentials, there is not a meaningful distinction between “multiplicative” and “additive” in this case; the equations describe the relationships better than such terms; we nonetheless call this model “Additive currencies.” Note also that these equations don’t incorporate absolute levels of the currencies, but rather how they interact with each other in the context of the trade-off. The absolute level is assumed to be constant and sufficient for survival; the *PTV* parameters describe not level of the resource, but which side of the trade-off is favoured in its allocation.

For the remaining 2 two-currency models (“Maximum currency” and “Minimum currency”), one of the two currencies dominates. This might equate to a biological situation in which the two processes are on a shared pathway, and only the more limiting or less limiting has an effect. For example, plant trade-offs between growth and reproduction might be mediated by both levels of energy (largely photosynthesis) and nitrogen intake [46]; when one of these two resources is scarce, it might completely determine the trade-off. For the Maximum currency model, the currency with the larger effect dominates. The governing equations are thus the same as Eqs (3) and (4) of the one-currency model, except that *PTV* is whichever currency has a larger local effect, i.e., *PTV* = max(|*PTV*_*1*_|,|*PTV*_*2*_|). In the Minimum currency model, only the currency with the smaller local effect dominates, min(|*PTV*_*1*_|,|*PTV*_*2*_|).

### Parameters

A summary of all model parameters is provided in Table 1. The model has a number of fixed parameters (*i*.*e*., those not allowed to vary except in sensitivity analyses in the SI, first eight in Table 1). Three key input parameters were fixed at the beginning of each simulation but allowed to vary across simulations: *W*_*1*_, *W*_*2*_, and *b*_*0*_. The output parameters—those that evolve within the model—are the values of the two physiological trade-off traits (*PTV*_*1*_ and *PTV*_*2*_), age at death (*A*_*d*_), fertility (*f*), and life reproductive success (*LRS*). The output parameters’ means were tracked across generations in each simulation for comparison. Our focus is particularly on the values of *PTV*_*1*_ and *PTV*_*2*_ to examine whether final physiological state and trade-off strategy change when multiple currencies exist, and on *LRS*, to quantify whether and how the co-existence of trade-off currencies may affect the ability of organisms to search fitness landscapes efficiently for optimal parameter combinations.

**Table 1:** Model parameters and their specifications.

Parameter	Symbol	Type*	Model	Theoretical Values	Value(s) used	Sensitivity analyses (Online supplement)	Figure
Gompertz coefficient	*a*	Fixed	All	[0,∞]	0.08	0.00001, 0.0001, 0.001, 0.01, 0.1, 0.2	S21
Heritability	*h*	Fixed	All	[0,1]	0.95	1, 0.99, 0.95, 0.9, 0.8, 0.5, 0.2	S16
Lag parameter in *b* function	γ	Fixed	All	[-∞,∞]	-2	-10, -7, -4, -2, -1, 0	S22
SD of baseline PTVs	-	Fixed	All	[0,∞]	0.1	0, 0.01, 0.1, 0.2, 0.5 1, 3	S13
SD of *LRS*	-	Fixed	All	[0,∞]	0.1 of *LRS*	0, 0.01, 0.1, 0.2, 0.5 1, 3	S15
Maximum fertility per unit time	*f*_*max*_	Fixed	All	[0,∞]	1	NA	
Individuals per generation	-	Fixed	All	-	10,000	NA	
Generations	-	Fixed	All	-	500	10,000	S12
Weight	*W*	VAS	1	[0,∞]	exp(N(0, 2))	NA	
Weight for *PTV*1	*W*_*1*_	VAS	2,3,4	[0,∞]	exp(N(0, 2))	NA	
Weight for *PTV*2	*W*_*2*_	VAS	2,3,4	[0,∞]	exp(N(0, 2))	NA	
Minimum possible aging rate	*b*_*0*_	VAS	All	[0,∞]	[0,1]	NA	
Physiological trait value	*PTV*	Evolving	1	[-∞,∞]	*N*(0, 0.1) & Eqn (S1)	NA	
Physiological trait value #1	*PTV*_*1*_	Evolving	2,3,4	[-∞,∞]	*N*(0, 0.1) & Eqn (S1)	NA	
Physiological trait value #2	*PTV*_*2*_	Evolving	2,3,4	[-∞,∞]	*N*(0, 0.1) & Eqn (S1)	NA	
Gompertz aging rate	*b*	Evolving	All	[0,∞]	Eqns (3) & (5)	NA	
Age at death	*A*_*d*_	Evolving	All	[0,∞]	see text	NA	
Fertility	*f*	Evolving	All	[0,∞]	Eqns (4) & (6)	NA	
Lifetime reproductive success	*LRS*	Evolving	All	[0,∞]	Eqn (2)	NA	

*There are three parameter types: those fixed across all simulations except sensitivity analyses ("Fixed"), those fixed within a given simulation but varying across simulations ("VAS"), and those that evolve across generations within each simulation ("Evolving"). Evolving parameters are tracked and used to assess what happens in a simulation, while VAS parameters are changed to assess model sensitivity to their values.

### Analyses presented

We systematically varied *W*_*1*_ and *W*_*2*_ across five values from low to high, creating a 5 × 5 matrix of results for different possible combinations of currency weights. The middle value was established by trial-and-error to identify a value of *W* for which *PTV* does not evolve in the one-currency model. As will be seen, the relative weights are very important, and this strategy allowed us to observe what happens when the weights are equal, or when one is much larger than the other. We also sampled parameter space randomly 1,000 times from the following distributions: *b*_*0*_ from the distribution given by *N*(0,0.2)^2^, and *W*_*1*_ and *W*_*2*_ from the log-normal distribution specified by *e*^*N*(0,2)^. Additional analyses examined the importance of stochasticity and contingency in this evolutionary framework by assessing the stability of results across multiple identical runs (100 per parameterisation), incorporated a linear increase in fertility with age (representing an impact of experience on fertility, for example), and extended the number of currencies from two to four. Extensive sensitivity analyses are presented in the SI.

## Supporting information

S1 Text Principal modelling challenges. (PDF)

S2 Text Sensitivity analysis framework. (PDF)

S3 Text Creating the initial population. (PDF)

S4 Text Creating subsequent generations. (PDF)

S5 Text Calculation of age at death. (PDF)

S6 Text Calculation of fertility and lifetime reproductive success. (PDF)

S7 Text Modelling trade-offs. (PDF)

S8 Text Discrete generations, stable population. (PDF)

S9 Text Number of generations. (PDF)

S10 Text Evolution of more than two trade-off currencies. (PDF)

S1 Fig Functional forms of (A) aging rate and (B) fertility trade-offs. For increasing values of the currency (*PTV*), increases in both the *b* parameter (i.e., faster aging) and increases in fertility (*f*) occur, but fertility benefits become asymptotic due to constraints (e.g. physiological) at *f*_*max*_. Likewise, *b* can only go but so low, as limited by *b*_*0*_. The constraints are both biologically realistic and necessary for the model to produce stable results at intermediate values of both *b* and fertility. In our models, the value used to calculate *b* or fertility is always based on the *PTV* multiplied or divided by its weight, *W* (see Eqs (3) and (4)).
(TIF)

S2 Fig Reproduction of Fig 1, but changing the parameter *γ* from Eq (3) to -10 such that range of important variation in aging rate (*b*, panel A) and fertility (*f*, panel B) is not aligned across values of *PTV*. (TIF)

S3 Fig Functional relationships between *PTV* values and aging rate (*b*, panel A) and fertility (*f*, panel B) based on alternative equations considered while developing the model, equations (S5) and (S6). (TIF)

S4 Fig Variation of results across 100 identical simulations where *W*_*1*_ = 0.43 and *W*_*2*_ = 0.1. The *x*-axis represents the mean value of the indicated trait for the 10,000 individuals in last of the 500 generations in each simulation (boxplots are mean, interquartile range, and 1.5 times interquartile range). The four models are indicated on the *y*-axis. Results are largely consistent from one simulation to the next, confirming the validity of our approach to use one simulation for each parameter combination rather than the average of many.
(TIF)

S5 Fig Results of 1,000 simulations generated by randomly sampling parameter space of the weights *W*_*1*_ and *W*_*2*_ and the rate of ageing *b*_*0*_. The three histograms show the respective differences between the final life time reproductive success *LRS* for each two-currency model and the final *LRS* for the one-currency model (positive indicating greater *LRS* in the two-currency scenario). The correlation plot represents correlations in model input parameters and results across the 1,000 runs. The shape and direction of the ellipses represent correlation strengths and directions, respectively, with correlation coefficients as indicated in the color bar (right). “*W* diff” indicates the difference in weights, specifically |log(*W1/W2*)|. This difference is strongly positively correlated with the values in the first histogram, and more weakly with the other two, implying that the additive model is increasingly effective at circumventing trade-offs as their relative effects on survival and reproduction differ more.
(TIF)

S6 Fig Changes in the evolution of the first physiological trait value, *PTV*_*1*_, under a variety of scenarios. *PTV*_*1*_ can be considered a physiological or behavioural trait with impacts on both fertility and survival. *b*_*0*_ is fixed at 0.01. Effects are largest when *W*_*1*_ = 1, but even under other scenarios there are clear differences; see S7 Fig for finer-scale y-axes.
(TIF)

S7 Fig As in S6 Fig but with a reduced y-axis range, to show smaller differences. (TIF)

S8 Fig Changes in the evolution of the second physiological trait value, *PTV*_*2*_, under a variety of scenarios. *PTV*_*2*_ can be considered a physiological or behavioural trait with impacts on both fertility and survival. *b*_*0*_ is fixed at 0.01. Effects are largest when *W*_*2*_ = 1, but even under other scenarios there are clear differences; see S9 Fig for finer-scale y-axes.
(TIF)

S9 Fig As in S8 Fig but with a reduced y-axis range, to show smaller differences. (TIF)

S10 Fig A model with four different trade-off currencies instead of two. The additive model is used for two-, three-, and four-currency models, with weights as specified in the figure. Note that while each model produces a different result, the order is important: W3 and W4 are respectively more extreme than any weights in the preceding models. If the order is reversed such that successive currency weights are found within the range of preceding currency weights, no difference is seen as more currencies are added (data not shown).
(TIF)

S11 Fig A run of the model incorporating a linear increase in fertility with age (fertility = age/3), reflecting, for example, the benefits of experience in reproduction. Average lifetime fertility (bottom left panel) becomes related to age at death, and consequently adds some stochasticity to this parameter relative to other models. Otherwise, results are qualitatively indistinguishable from other models.
(TIF)

S12 Fig A model extending to 10,000 generations instead of 500. Note that convergence of life history traits is reached relatively early, suggesting that our 500-generation models are largely sufficient. Also note that physiological trait values are not stable in models with multiple currencies and can vary without much impact on life-history traits.
(TIF)

S13 Fig Sensitivity analyses of the standard deviation of baseline *PTV*s. We ran 100 simulations in which this standard deviation was fixed at one of the values along the *x*-axis. For each of the four models in each simulation, we present the average trait value at the 500^th^ (final) generation on the *y*-axis. *b*_*0*_ was fixed at 0.01, *W*_*1*_ at 0.43, and *W*_*2*_ at 0.1. Note that some initial variation is necessary for the model to run well, but given that heritability < 1, this variation can be minimal and model will still produce reasonable results.
(TIF)

S14 Fig Functional relationship between mean age at death *A*_*d*_ and aging rate *b* based on a non-linear model fitting the mean to the values of *b* used to simulate age at death. (TIF)

S15 Fig Sensitivity analyses of the stochastic coefficient of variation (*SCV*) of *LRS*. This parameter is the factor by which expected *LRS* is multiplied to generate the standard deviation of actual *LRS*: each individual’s *LRS* is sampled from the distribution *LRS* ~ *N*(*f × A*_*d*_, *f × A*_*d*_ *× SCV*) (Eq (2)).We ran 100 simulations in which this standard deviation was fixed at one of the values along the *x*-axis. For each of the four models in each simulation, we present the average trait value at the 500^th^ (final) generation on the *y*-axis. *b*_*0*_ was fixed at 0.01, *W*_*1*_ at 0.43, and *W*_*2*_ at 0.1. We use *SCV* = 0.1 in our simulations, but it can be seen here that the model is not sensitive to values in a wide range around this.
(TIF)

S16 Fig Sensitivity analyses of the heritability parameter. We ran 100 simulations in which heritability was fixed at one of the values along the *x*-axis. For each of the four models in each simulation, we present the average trait value at the 500^th^ (final) generation on the *y*-axis. *b*_*0*_ was fixed at 0.01, *W*_*1*_ at 0.43, and *W*_*2*_ at 0.1. Note that when heritability is too high, evolution is very slow and constrained to the initial parameter range; when it is too low, stochasticity dominates. The strongest signal is thus when heritability is between 0.8 and 0.95. We use 0.95 in our models.
(TIF)

S17 Fig A simulation using a Gompertz mortality function with the trade-off acting on the *a* parameter to generate age-specific risk of death. The *b* parameter was fixed at 0.1. Compare to Fig 2 (Gompertz function, trade-off acting on the *b* parameter) to see that results are not sensitive to mortality function specification.
(TIF)

S18 Fig A simulation using a Gompertz-Makeham mortality function with the trade-off acting on the *a* parameter to generate age-specific risk of death. The *b* parameter was fixed at 0.09 and the *c* parameter was fixed at 0.01. Compare to Fig 2 (Gompertz function, trade-off acting on the *b* parameter) to see that results are not sensitive to mortality function specification.
(TIF)

S19 Fig A simulation using a Gompertz-Makeham mortality function with the trade-off acting on the *b* parameter to generate age-specific risk of death. The *a* parameter was fixed at 0.07 and the *c* parameter was fixed at 0.01. Compare to Fig 2 (Gompertz function, trade-off acting on the *b* parameter) to see that results are not sensitive to mortality function specification.
(TIF)

S20 Fig A simulation using a simple linear mortality function with the trade-off acting on the slope to generate age-specific risk of death. The intercept was fixed at 0.08. Compare to Fig 2 (Gompertz function, trade-off acting on the *b* parameter) to see that results are not sensitive to mortality function specification.
(TIF)

S21 Fig Sensitivity analyses of the Gompertz coefficient *a*. We ran 100 simulations in which *a* was fixed at one of the values along the *x*-axis. For each of the four models in each simulation, we present the average trait value at the 500^th^ (final) generation on the *y*-axis. *b*_*0*_ was fixed at 0.01, *W*_*1*_ at 0.43, and *W*_*2*_ at 0.1. Because lifespan increases as *a* decreases, we scaled lifetime reproductive success (*y*-axis of the final panel) to the value in the single currency model. We used *a* = 0.08 throughout our analyses, but it can be seen here that model conclusions do not depend heavily on this parameter, and that in fact smaller values (which may be more realistic) would accentuate our findings.
(TIF)

S22 Fig Sensitivity analyses of the lag parameter γ in Eqs (3) and (5). We ran 100 simulations in which γ was fixed at one of the values along the *x*-axis. For each of the four models in each simulation, we present the average trait value at the 500^th^ (final) generation on the *y*-axis. *b*_*0*_ was fixed at 0.01, *W*_*1*_ at 0.43, and *W*_*2*_ at 0.1. Note that we stopped the *x*-axis at γ = 0 because for higher values the model often crashed: aging rate becomes so high that life expectancy goes to zero even with essentially zero fertility. As shown in S1 Fig, the key for a meaningful analysis is that γ align the key regions of variation in the fertility and aging rate functions; while extreme values are clearly problematic, this figure shows that meaningful results can be obtained at γ = -2 and for a substantial range around this value. As shown in the last panel, γ = -2 seems to be a sweet spot where the performance of the four models is most distinct.
(TIF)

S23 Fig A replication of Fig 3 showing model outcomes across values of the W parameters, but based on equations (S5) and (S6). As can be seen, changing the trade-off equations has little impact on the qualitative model results in this case.
(TIF)

S1 File Main model. (R)

S2 File Source functions for main model. (R)

S3 File Multiple currencies model. (R)

S4 File Source functions for multiple currencies model. (R)

S5 File Repeated model. (R)
