# Supplementary material for: Co-existence of multiple trade-off currencies shapes evolutionary outcomes
Source: PLoS One. 2017 Dec 7;12(12):e0189124. doi: 10.1371/journal.pone.0189124 (PMC5720690; doi:10.1371/journal.pone.0189124)
Supplement: S1 Text — (PDF) [file pone.0189124.s001.pdf]

Co-existence of multiple trade-off currencies has major impacts on evolutionary outcomes

Alan A. Cohen, Caroline Isaksson, and Roberto Salguero-Gómez

Details on model parameterisation and results

The results of a model of the sort we are presenting here depend heavily on the particular specifications, and our ability to present all the details of model development, results, and sensitivity analyses is limited in a normal-length article. In ten Supporting Information sections, we present details of our reasoning, parameter specification, and relevant results. We do so in sections based on key aspects of model structure and parameterisation.

S1 Text. Principal modelling challenges

The principal modelling challenges encountered were: (1) Both reproduction and lifespan need to be free to evolve, creating the possibility for population crashes or explosions. We control this by keeping population size fixed. (2) Lifespan and reproduction need to be linked by a trade-off, and a function is thus necessary to describe the trade-off. Obviously, the real biological function is largely unknown. We show that the precise form of the function does not affect conclusions. (3) Weights for the currencies are necessary to avoid the trivial case where multiple currencies reduce mathematically to the equivalent of one. The weights must also then be built into the functions. (4) While not absolutely necessary (indeed, we explore alternatives), it is desirable to have mortality follow a known demographic process such as a Gompertz function. This implies another layer of complexity where the trade-off does not act directly on mortality, but on a parameter of a mortality function.
