# Supplementary material for: Co-existence of multiple trade-off currencies shapes evolutionary outcomes
Source: PLoS One. 2017 Dec 7;12(12):e0189124. doi: 10.1371/journal.pone.0189124 (PMC5720690; doi:10.1371/journal.pone.0189124)
Supplement: S2 Text — (PDF) [file pone.0189124.s002.pdf]

Co-existence of multiple trade-off currencies has major impacts on evolutionary outcomes

Alan A. Cohen, Caroline Isaksson, and Roberto Salguero-Gómez

Details on model parameterisation and results

The results of a model of the sort we are presenting here depend heavily on the particular specifications, and our ability to present all the details of model development, results, and sensitivity analyses is limited in a normal-length article. In ten Supporting Information sections, we present details of our reasoning, parameter specification, and relevant results. We do so in sections based on key aspects of model structure and parameterisation.

S2 Text. Sensitivity analysis framework

For most parameters on which we conducted sensitivity analyses (Table 1), we did so by choosing a set of six or seven plausible values representing a wide range of possibilities. We then fixed all other parameters to the defaults in Table 1, including b_0 = 0.01, W_1 = 0.43, and W_2 = 0.1. We ran 100 iterations of the model in which each possible value for the parameter was chosen an approximately equal number of times. We then plotted the final mean values (500th generation) for each of the five evolving traits against the potential values of the parameter in question.

In some cases, this approach was not feasible. For example, both mortality and trade-offs required specification of a function rather than a parameter. In these cases, we examined a figure structured like Fig. 3 to see if there were important qualitative differences across potential functions. Likewise, for number of generations, we compared a single run to 10,000 generations with Fig. 3. Specifics are given below as necessary.
