# Supplementary material for: Co-existence of multiple trade-off currencies shapes evolutionary outcomes
Source: PLoS One. 2017 Dec 7;12(12):e0189124. doi: 10.1371/journal.pone.0189124 (PMC5720690; doi:10.1371/journal.pone.0189124)
Supplement: S3 Text — (PDF) [file pone.0189124.s003.pdf]

Co-existence of multiple trade-off currencies has major impacts on evolutionary outcomes

Alan A. Cohen, Caroline Isaksson, and Roberto Salguero-Gómez

Details on model parameterisation and results

The results of a model of the sort we are presenting here depend heavily on the particular specifications, and our ability to present all the details of model development, results, and sensitivity analyses is limited in a normal-length article. In ten Supporting Information sections, we present details of our reasoning, parameter specification, and relevant results. We do so in sections based on key aspects of model structure and parameterisation.

S3 Text. Creating the initial population

Each generation had the same number of individuals (10,000), equivalent to assuming a population at carrying capacity with probability of recruitment into the population being random with respect to individual traits. In other words, we assume that all fitness-related traits are captured in our fertility and age-at-death measures, and in the trade-offs between them. For any given individual in a simulation, only two independent parameters are necessary: the two currency trait values, PTV_1 and PTV_2 (or just *PTV* in the case of the single currency model). PTV_1 and PTV_2 can be considered as two independent physiological (or behavioural) traits each of which acts on reproduction and survival through a trade-off. For example, we might consider PTV_1 to be an allocation strategy for energy that could be invested in either egg production or DNA repair and PTV_2 to be an allocation strategy for carotenoids that could be invested either in membrane stabilization and antioxidant defences, or in secondary sexual traits such as plumage color. High values of the traits indicate greater investment in reproduction; lower values indicate greater investment in survival. Once PTV_1 and PTV_2 are known, fertility, aging rate, age at death, and lifetime reproductive success can be generated (see below). We thus describe here how we generate these two values, describing the others below.

We generated random values of each *PTV* for the first generation using a normal distribution centered at zero and with a standard deviation fixed at 0.1, $PTV \sim N(0, 0.1)$. We ran sensitivity analyses on this standard deviation, in particular because greater initial variation might accelerate evolution under certain parameterisations. S3 Fig. shows that, while qualitative conclusions

change minimally for different values of this standard deviation, large values amplify the differences between the additive model and the others. When this standard deviation is 0 (no variation among individuals), the model does not perform well, likely because our default heritability is 0.95, very high. The standard deviation value used, 0.1, was chosen as an appropriate balance between having sufficient variation for the model to evolve well and not exaggerating any potential effects.
