# Supplementary material for: Co-existence of multiple trade-off currencies shapes evolutionary outcomes
Source: PLoS One. 2017 Dec 7;12(12):e0189124. doi: 10.1371/journal.pone.0189124 (PMC5720690; doi:10.1371/journal.pone.0189124)
Supplement: S4 Text — (PDF) [file pone.0189124.s004.pdf]

Co-existence of multiple trade-off currencies has major impacts on evolutionary outcomes

Alan A. Cohen, Caroline Isaksson, and Roberto Salguero-Gómez

Details on model parameterisation and results

The results of a model of the sort we are presenting here depend heavily on the particular specifications, and our ability to present all the details of model development, results, and sensitivity analyses is limited in a normal-length article. In ten Supporting Information sections, we present details of our reasoning, parameter specification, and relevant results. We do so in sections based on key aspects of model structure and parameterisation.

S4 Text. Creating subsequent generations

In order to create inheritance of the *PTV* values and to preserve information on individual fitness, each subsequent generation was created from the previous one by sampling, with replacement, the *PTV* values of the 10,000 individuals of the previous generation, probabilistically weighted to each individual's *LRS*. The probability weights were the *LRS* of each individual divided by the total reproductive success of the generation. This method is equivalent to randomly sampling all offspring from the previous generation to create a new population of 10,000. Additionally, we incorporated a stochastic element in the transmission of *PTV* values across generations, equivalent to imperfect genetic transmission or control of the trait. This was represented by a heritability parameter, *h*. Actual *PTV* values in the subsequent generation (PTV_a) were thus calculated based on the sampled *PTV* (PTV_s) values as follows:

$$PTV_a \sim N(PTV_s, PTV_s \times (1\text{-}h)) \qquad \text{(S1)}$$

This equation means that *h* is overall fidelity in transmission of traits across generations; loss of fidelity could be environmental (as with heritability), but could also represent mutation, etc. *h* was fixed at 0.95. We used sensitivity analyses to explore values of *h* between 0.2 and 1. S9 Fig. shows that *h* is optimised between 0.8 and 0.95, particularly for the additive model. This is to be expected, since *h* as we have defined it can limit evolution when it is very close to 1, particularly when the initial *PTV* variance is low, as in our model (0.1). Low values of *h* cause a loss of signal

from one generation to the next, and are thus expected to slow evolution. In this sense, *h* and the initial *PTV* variance are parameters that have less impact on model conclusions; optimising them is about ensuring that there is sufficient heritable variation for selection to act. Our choice of parameter values thus reflects a supposition of our model: that the traits in question have substantial heritable variation, a *sine qua non* for exploring the questions we are posing. Optimisation also accelerates evolution, reducing the number of generations needed and thus increasing computational efficiency. Results of these sensitivity analyses also confirm that the qualitative effects are reproduced under different amounts of heritable variation, but it is not clear whether the more limited magnitude of effects with less heritable variation is due to slower evolution or to less evolutionary potential.
