# Supplementary material for: Co-existence of multiple trade-off currencies shapes evolutionary outcomes
Source: PLoS One. 2017 Dec 7;12(12):e0189124. doi: 10.1371/journal.pone.0189124 (PMC5720690; doi:10.1371/journal.pone.0189124)
Supplement: S5 Text — (PDF) [file pone.0189124.s005.pdf]

Co-existence of multiple trade-off currencies has major impacts on evolutionary outcomes

Alan A. Cohen, Caroline Isaksson, and Roberto Salguero-Gómez

Details on model parameterisation and results

The results of a model of the sort we are presenting here depend heavily on the particular specifications, and our ability to present all the details of model development, results, and sensitivity analyses is limited in a normal-length article. In ten Supporting Information sections, we present details of our reasoning, parameter specification, and relevant results. We do so in sections based on key aspects of model structure and parameterisation.

S5 Text. Calculation of age at death

Age at death for each individual was generated stochastically from a probability distribution based on increasing mortality with age. The rate of this increase (aging rate, b) was determined by the trade-off functions (see "*Modelling trade-offs*" in the main text and S7 Text). We compared five functions linking b to the probability distribution. The simplest was a linear increase in mortality with age with slope b:

$$\mu(x) = a + bx \tag{S2}$$

where $\mu(x)$ is mortality at age x and a is a constant (fixed at 0.08). We also used the Gompertz model [1] or the Gompertz-Makeham model [e.g. 2], respectively:

$$\mu(x) = a \times e^{bx} \tag{S3}$$

$$\mu(x) = a \times e^{bx} + c \tag{S4}$$

where $\mu(x)$ is mortality at age x, a is initial mortality, b is the rate of exponential increase in mortality, and c is an age-independent mortality component present in the Gompertz-Makeham (S4) but not the Gompertz model (S3). For each of these two models we allowed the trade-off to act on either a or b. All five models produced results that were qualitatively nearly identical (Fig.

3 and S10-S13 Figs); we chose the Gompertz model with the trade-off acting on *b* because it was computationally much more efficient than the Gompertz-Makeham in our modeling framework and the interpretation of *b* as an aging rate is clear. Variation in *b* goes against some literature suggesting that *b* is constant across human populations [3], but is desirable from a perspective of modeling a trade-off affecting aging rate and is consistent with variation in *b* across species; in any case the choice of model does not affect our conclusions.

Because our final retained model was equation (1) with the trade-off acting on *b*, we needed a fixed value of *a*, chosen as 0.08, within the range of observed natural variation observed [4-6]. We conducted sensitivity analyses on the values of *a* given in Table 1. S14 Fig. shows that variation in *a* has little impact on conclusions. Note that for the *LRS* panel, we have scaled the *y*-axis by dividing by the value for the single currency model. This is necessary because changes in *a* create major differences in lifespan and thus *LRS*. Scaling allows us to see that the relative effects are stable.

References

1. Gompertz B. On the Nature of the Function Expressive of the Law of Human Mortality, and on a New Mode of Determining the Value of Life Contingencies. Philosophical Transactions of the Royal Society of London. 1825;115(ArticleType: research-article / Full publication date: 1825 / Copyright © 1825 The Royal Society):513-83.
2. Pletcher. Model fitting and hypothesis testing for age-specific mortality data. Journal of Evolutionary Biology. 1999;12(3):430-9. doi: 10.1046/j.1420-9101.1999.00058.x.
3. Vaupel JW. Biodemography of human ageing. Nature. 2010;464(7288):536-42.
4. de Magalhães JP, Cabral JAS, Magalhães D. The Influence of Genes on the Aging Process of Mice: A Statistical Assessment of the Genetics of Aging. Genetics. 2005;169(1):265-74. doi: 10.1534/genetics.104.032292.
5. Pletcher SD. Model fitting and hypothesis testing for age-specific mortality data. Journal of Evolutionary Biology. 1999;12(3):430-9.
6. Simons MJ, Koch W, Verhulst S. Dietary restriction of rodents decreases aging rate without affecting initial mortality rate–a meta-analysis. Aging Cell. 2013;12(3):410-4.
