# Supplementary material for: Co-existence of multiple trade-off currencies shapes evolutionary outcomes
Source: PLoS One. 2017 Dec 7;12(12):e0189124. doi: 10.1371/journal.pone.0189124 (PMC5720690; doi:10.1371/journal.pone.0189124)
Supplement: S6 Text — (PDF) [file pone.0189124.s006.pdf]

Co-existence of multiple trade-off currencies has major impacts on evolutionary outcomes

Alan A. Cohen, Caroline Isaksson, and Roberto Salguero-Gómez

Details on model parameterisation and results

The results of a model of the sort we are presenting here depend heavily on the particular specifications, and our ability to present all the details of model development, results, and sensitivity analyses is limited in a normal-length article. In ten Supporting Information sections, we present details of our reasoning, parameter specification, and relevant results. We do so in sections based on key aspects of model structure and parameterisation.

S6 Text. Calculation of fertility and lifetime reproductive success

Fertility per unit time was assumed to be constant across the lifespan and was constrained to be non-negative and below a physiological constraint threshold, f_{max}, fixed at 1 in all our models. Because our model keeps population size constant, changing f_{max} simply changes the scale of the fertility variable (verified empirically). What is important here are the relative fertility contributions, not the absolute scale. Each individual's fertility value (*i.e.*, biological reproductive potential per unit time, based on the trade-off) varied depending on the individual's specific trade-off currency trait values.

LRS for each individual was calculated using equation (2) in the main text. In order to introduce a stochastic component to the model, reflecting actual variability in year-to-year reproductive success, we drew an individual's *LRS* from a normal distribution centered around the expected value, $f \times A_d$. Because the scales of f and A_d varied across models for other reasons (e.g., the mortality function and parameters used), it made sense to scale the variance to expected *LRS*. To do this, we simply multiplied expected *LRS* by a constant, 0.1, to generate the standard deviation of the distribution. We call this the stochastic coefficient of variation (*SCV*). We ran sensitivity analyses examining different values of *SCV*. S8 Fig. shows that almost all evolving traits show minimal differences across values of *SCV*, with the exception that *LRS* increases when the *SCV* is very high. Even this increase is absolute, not relative across the four models.
