# Supplementary material for: Co-existence of multiple trade-off currencies shapes evolutionary outcomes
Source: PLoS One. 2017 Dec 7;12(12):e0189124. doi: 10.1371/journal.pone.0189124 (PMC5720690; doi:10.1371/journal.pone.0189124)
Supplement: S7 Text — (PDF) [file pone.0189124.s007.pdf]

Co-existence of multiple trade-off currencies has major impacts on evolutionary outcomes

Alan A. Cohen, Caroline Isaksson, and Roberto Salguero-Gómez

Details on model parameterisation and results

The results of a model of the sort we are presenting here depend heavily on the particular specifications, and our ability to present all the details of model development, results, and sensitivity analyses is limited in a normal-length article. In ten Supporting Information sections, we present details of our reasoning, parameter specification, and relevant results. We do so in sections based on key aspects of model structure and parameterisation.

S7 Text. Modelling trade-offs

An individual's *PTV* value can be thought of as a trait, say physiological, that determines where the individual is situated on the high fertility-short lifespan to low fertility-long lifespan spectrum [1]. For example, the *PTV* might indicate how much energy or specific nutrients the individual invests in physiological maintenance at the expense of short-term reproduction. *PTV* values thus vary across individuals within each simulation, but are fixed across an individual's lifespan.

In addition to the *PTV*, it is necessary to have a parameter representing currency weight (*W*), which characterizes the relative gains that an individual can expect in fertility for each unit loss in lifespan, and *vice versa*. The weight does not vary across individuals, and it is assumed constant within each simulation. Figure 1 describes weights in detail. Any given trade-off currency (*PTV*s in Fig 1A) describes a negative relationship between two or more fitness components (survival and fertility here). Some currencies may be more constraining than others (PTV_1 versus PTV_2 in Fig 1A). Others may have different slopes (PTV_3 in Fig 1A), indicating different weights. Note that the conceptual representation in Fig. 1A is linear, but the actual relationships in our model (and likely reality) have no reason to be linear as long as they are monotonically negative. The actual forms are shown in Fig 1D, overlayed on a heatmap of expected LRS. Note that selection will move the *PTV*s along the curves determined by their weights, searching for the value that maximizes expected LRS. However, not all curves pass through the same maximal LRS, implying that some weights are associated with higher fitness than others. This is why our Minimum and Maximum models differ from the single-currency model.

As a concrete example of why weights are necessary, let us consider again the example of carotenoids as sexual signals described in the introduction. In birds, carotenoids are irreversibly deposited into the feather during feather growth and maturation. In animals with ornamented skin, such as reptiles, carotenoids must be constantly supplied to the skin to maintain the colour. Depending on whether skin pigmentation is maintained constantly, the carotenoid cost of skin pigmentation might thus be substantially higher than feather pigmentation [2, but see 3]. Accordingly, a species that uses carotenoids in a few small feathers, such as a European greenfinch (*Chloris chloris*, Carduelis) might much more efficiently improve reproductive success by shunting carotenoids to these feathers than a species that uses extensive skin pigmentation, like painted dragons (*Ctenophorus pictus,* Agamidae). The skin-pigmented species might thus evolve further toward the live slow-die old end of the spectrum, whereas the feather pigmented species would evolve toward the live fast-die young end. These differences in the relative reproduction and survival gains as each trait varies are captured by the weights. Although this is example is somewhat hypothetical, such variation in cost-benefits of currencies undoubtedly exists.

An understanding of weights is crucial to an understanding of our model, and we thus offer an analogy (recapitulated in the Fig 5 legend). Consider a bank that offers Reward Points (RPs) for various types of activity (e.g. credit card usage). The bank may allow you the option of using these points to purchase goods online (let's call that Spending) or to invest in specific savings/stock funds (we'll call that Savings). Presumably, you are also using your regular money ($, not RPs) to do some Spending and some Savings. However, there is no guarantee that the bank will make the buying power of RPs proportionately equivalent to that of money. That is, the bank may give you a better (or worse) deal on Savings relative to Spending if you use your RPs rather than money (e.g. 1 RP = $1 for Spending, 1 RP = $1.50 for Savings). Savings versus Spending is the trade-off (like Survival versus Reproduction). RPs and money are two monetary currencies, equivalent to energy and carotenoids. The weights in each case are the relative buying power, potentially different between currencies (e.g. 1 and 1.5 respectively), that indicate which currency is more profitably invested to maximize which side of the trade-off.

The principles underlying our choice of trade-off functions, as well as the functions themselves, are presented in the main text. Here we present the logic behind our choices as well as sensitivity analyses on parameters within the functions and on other potential functions. The largest challenge in designing trade-off functions was to arrive at functions that produce trade-offs that are

biologically realistic, both in terms of results and in terms of underlying assumptions. We knew our key input parameters (PTV_1, PTV_2, W_1, W_2) and needed to design models that would properly respond to changes in these parameters. The most crucial aspect here was producing important variation in both age at death and fertility in the same range of *PTV* values. For example, S4 Fig shows the functional forms of the two final equations (3) and (4) relating *PTV*s to *b* and *f*. Included in equation (3) is the parameter γ, which is simply a lag in *PTV* that serves to align S4 Fig (panels A-B). Without this, crucial variation in *PTV* that produces changes in *b* produces no meaningful change in *f*, and *vice versa*. For this reason, γ was fixed at -2. S5 Fig shows what would happen with an inappropriate lag (γ = -10). S15 Fig shows behaviour of the model under various values of γ.

It was also critical that the model be constrained to an appropriate balance between fertility and survival. In many scenarios initially considered, one of the two went very close to zero and the other approached infinity, producing biologically implausible results. To ensure optima that were within reasonable ranges, we needed diminishing returns on fertility and survival as the *PTV* moved too far in the relevant direction. This was achieved by introducing the parameters b_0 and f_{max}. The way these parameters were introduced ensured asymptotically diminishing returns for both survival and fertility, and thus that the model would not "run away" in either direction. This is also biologically realistic, as both fertility and aging rate are likely to be constrained by broader processes than immediate trade-offs (e.g., there are physiological reasons birds cannot lay 10,000 eggs per clutch, regardless of selection pressure).

That said, we considered a wide range of functional forms for equations (3-6). For example:

$$b = b_0 \cdot \left(1 - \frac{1}{e^{e^{PTV/W}}}\right) \tag{S5}$$

and

$$f = \frac{f_{max}}{e^{e^{-PTV \cdot W}}} \tag{S6}$$

which produce the functional forms shown in S6 Fig. Equation S5 reflects an erroneous specification early in model construction: b_0 must be added, not multiplied, to the term in

parentheses for its interpretation as a minimal mortality level to hold. Nonetheless, we conducted many simulations before catching this error, and the results were largely unchanged from our final model (e.g. S23 Fig). In this sense, our model was surprisingly robust to the details of specification, as long as the specification produced minimally biologically plausible results. The example in S23 Fig is one of many alternative specifications that gave largely similar results but were eventually discarded for one reason or another, generally to keep the biological interpretation of the parameters reasonable.

Another challenge in specifying these models was that b and expected age at death (A_d) have a non-linear relationship (S7 Fig). Accordingly, the functional form in S4 Fig (panel A) does not represent the relationship between PTV and expected A_d. Close to zero, small changes in b have a large effect on A_d. Further from zero, changes in b have little effect. This is the reason we used the distribution $N(0,0.2)^2$ to sample values of b for our random exploration of parameter space: this distribution ensures higher density close to zero and lower density further.

Finally, even within the final equation for b (equation (3)), we considered a range of parameters to adjust the shape of the relationship. The full equation is thus:

$$b = b_0 + \alpha \cdot e^{\beta \cdot \frac{PTV+\gamma}{W}} \qquad \text{(S7)}$$

where α, β, and γ are shape parameters. We ran a large number of simulations testing different values of these parameters, but none of these would have changed our conclusions. For reasons of space, we do not include all these simulations here, but they can be easily run from our code, available here, which retains these parameters. In the end, we fixed α and β at 1 (thus simplifying our presentation by eliminating them). Nonetheless, it is worth noting that α functions essentially as a scale parameter defining the range of variation in b, and β functions as a shape parameter, with larger values producing a faster exponential increase. The function shown in S4 Fig (panel A) appears at first glance to have too abrupt an increase in b and thus seems to require a value of $\beta < 1$, but actually works well due to the non-linear relationship between b and A_d mentioned above (S7 Fig).

Equations (3) – (6) thus have only two fixed parameters that could be varied: γ and f_{max}. As noted above, f_{max} has no effect other than to control the scale of fertility (all changes in model results are directly proportional). We do show sensitivity analyses on γ, which is an important

parameter due its ability to align equations (3) and (4) (S15 Fig). While the higher *LRS* obtained under the additive model is never in question, the relative performances of the minimal, maximal, and single currency models do depend on γ, and there are major differences in results across γ values. Values of γ higher than 0 could not be modeled stably because they force very fast aging to be combined with very low reproduction and lead to model crashes in many simulations. As can be seen in the monotonic decrease in fertility, age at death, and *LRS* with increases in γ, higher γ forces tighter and tighter trade-offs; at low γ, high fertility can co-exist with high survival. We thus believe that there is a solid justification for using γ = -2 (or something close): when γ is much lower the trade-off is much weaker and we are no longer asking the same question, and when it is much higher the evolutionary options become constrained to the point of threatening population viability.

References

1. Stearns SC. The Evolution of Life Histories. New York: Oxford University Press; 1992.
2. Negro JJ, Bortolotti GR, Tella JL, Fernie KJ, Bird DM. Regulation of integumentary colour and plasma carotenoids in American Kestrels consistent with sexual selection theory. Functional Ecology. 1998;12(2):307-12. doi: 10.1046/j.1365-2435.1998.00176.x.
3. Simons MJ, Maia R, Leenknegt B, Verhulst S. Carotenoid-dependent signals and the evolution of plasma carotenoid levels in birds. Am Nat. 2014;184:741-51.
