# Supplementary material for: Co-existence of multiple trade-off currencies shapes evolutionary outcomes
Source: PLoS One. 2017 Dec 7;12(12):e0189124. doi: 10.1371/journal.pone.0189124 (PMC5720690; doi:10.1371/journal.pone.0189124)
Supplement: S8 Text — (PDF) [file pone.0189124.s008.pdf]

Co-existence of multiple trade-off currencies has major impacts on evolutionary outcomes

Alan A. Cohen, Caroline Isaksson, and Roberto Salguero-Gómez

Details on model parameterisation and results

The results of a model of the sort we are presenting here depend heavily on the particular specifications, and our ability to present all the details of model development, results, and sensitivity analyses is limited in a normal-length article. In ten Supporting Information sections, we present details of our reasoning, parameter specification, and relevant results. We do so in sections based on key aspects of model structure and parameterisation.

S8 Text. Discrete generations, stable population

Initial versions of the model did not constrain population size. While biologically more realistic, it was very hard to parameterise such models so as to avoid both extinction and unlimited, unrealistic exponential growth. Since population dynamics were not the focus of our simulations, we decided to use the model described in the main text, which resamples the individuals in the previous generation weighted by their *LRS* to create a subsequent generation of the same size. While not ideal, this solution does permit good approximation of relative fitness within each generation. The largest potential problem is that making generations discrete eliminates the selective advantage for short generation times, and thus biases the model toward favoring more survival and less fertility. Nonetheless, our objective was not to estimate the true value of *W*, and there is no reason to think that the advantage of multiple currencies would be affected by this bias.
