# Supplementary material for: Co-existence of multiple trade-off currencies shapes evolutionary outcomes
Source: PLoS One. 2017 Dec 7;12(12):e0189124. doi: 10.1371/journal.pone.0189124 (PMC5720690; doi:10.1371/journal.pone.0189124)
Supplement: S9 Text — (PDF) [file pone.0189124.s009.pdf]

Co-existence of multiple trade-off currencies has major impacts on evolutionary outcomes

Alan A. Cohen, Caroline Isaksson, and Roberto Salguero-Gómez

Details on model parameterisation and results

The results of a model of the sort we are presenting here depend heavily on the particular specifications, and our ability to present all the details of model development, results, and sensitivity analyses is limited in a normal-length article. In ten Supporting Information sections, we present details of our reasoning, parameter specification, and relevant results. We do so in sections based on key aspects of model structure and parameterisation.

S9 Text. Number of generations

S1 Fig. extends the number of generations to 10,000. It is clear that life-history traits converge very early, suggesting that the 500 generations used in the bulk of our models are largely sufficient. However, in the two currency models the physiological trait values can evolve even when the life-history trait values are stable. This supports the inference of an important stochastic component in physiological evolution (see Discussion).
