# Supplementary material for: Co-existence of multiple trade-off currencies shapes evolutionary outcomes
Source: PLoS One. 2017 Dec 7;12(12):e0189124. doi: 10.1371/journal.pone.0189124 (PMC5720690; doi:10.1371/journal.pone.0189124)
Supplement: S10 Text — (PDF) [file pone.0189124.s010.pdf]

Co-existence of multiple trade-off currencies has major impacts on evolutionary outcomes

Alan A. Cohen, Caroline Isaksson, and Roberto Salguero-Gómez

Details on model parameterisation and results

The results of a model of the sort we are presenting here depend heavily on the particular specifications, and our ability to present all the details of model development, results, and sensitivity analyses is limited in a normal-length article. In ten Supporting Information sections, we present details of our reasoning, parameter specification, and relevant results. We do so in sections based on key aspects of model structure and parameterisation.

S10 Text. Evolution of more than two trade-off currencies

We ran a small number of models tackling the question of how more than two trade-off currencies might interact in a similar framework in which the additive model was extended. S21 Fig. is an example. Broadly speaking, we found that results were determined by whatever two currencies had the most extreme weight (W) values. For example, if weights were 0.1, 1, and 2, the model results were largely determined by the two traits with weights 0.1 and 2; results would not change if we added another trait with weight 0.3. We believe this represents the fact that the trade-off we model is one-dimensional (between fertility and survival), and thus that two currencies with different weights can optimize the ability to capitalize in both directions along the axis. If this is correct, then the main impact of increasing the number of trade-off currencies beyond 2 in a one-dimensional trade-off framework is to increase the probability of having more extreme weights. However, if the true framework is not one-dimensional (e.g., if growth were considered a separate factor to trade off with survival and fertility), the number of key currencies could be larger than two (Fig. 5).
