# Supplementary figures and images for: Co-existence of multiple trade-off currencies shapes evolutionary outcomes
Source: PLoS One. 2017 Dec 7;12(12):e0189124. doi: 10.1371/journal.pone.0189124 (PMC5720690; doi:10.1371/journal.pone.0189124)

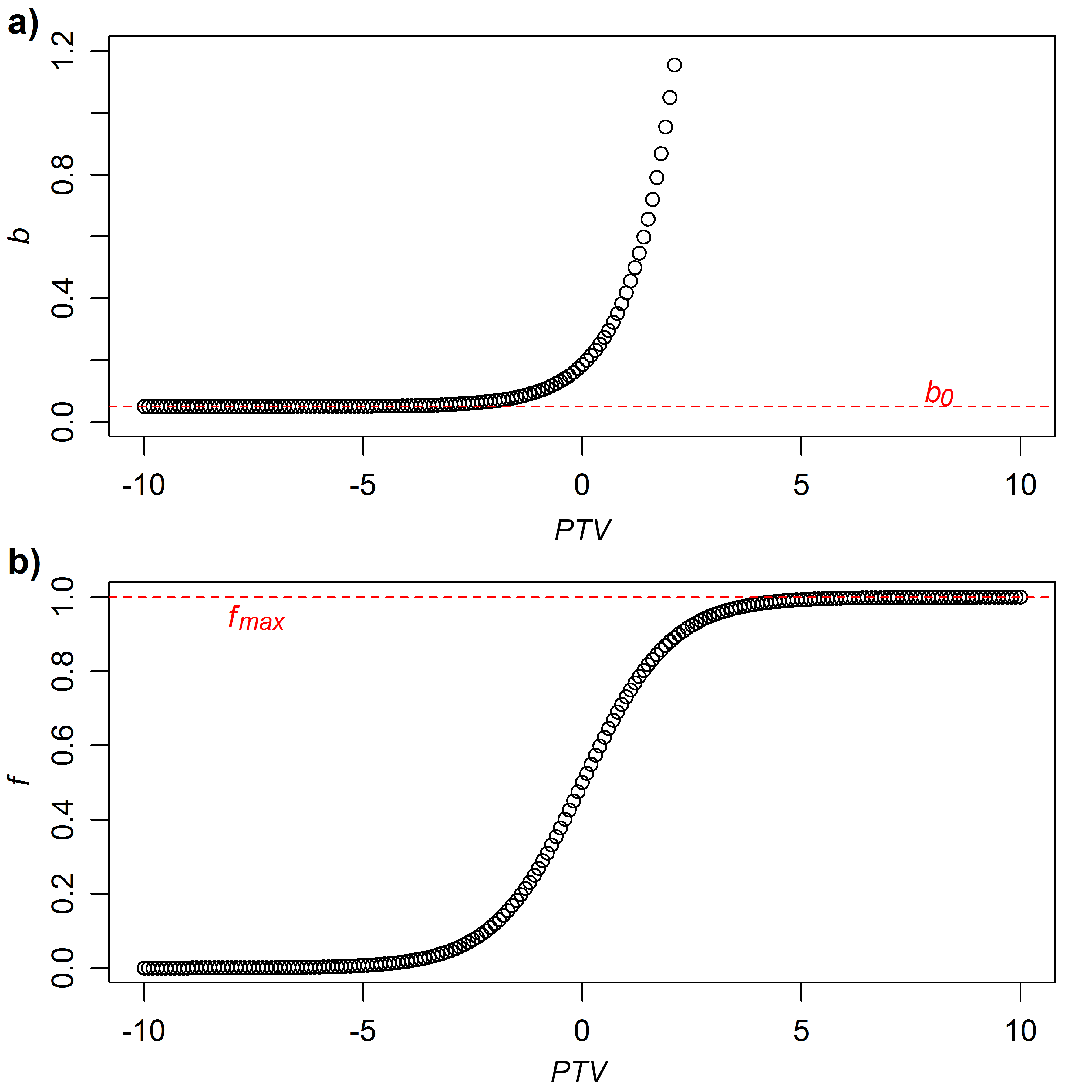

Supplement: S1 Fig — For increasing values of the currency (PTV), increases in both the b parameter (i.e., faster aging) and increases in fertility (f) occur, but fertility benefits become asymptotic due to constraints (e.g. physiological) at fmax. Likewise, b can only go but so low, as limited by b0. The constraints are both biologically realistic and necessary for the model to produce stable results at intermediate values of both b and fertility. In our models, the value used to calculate b or fertility is always based on the PTV multiplied or divided by its weight, W (see Eqs (3) and (4)). (TIF) [file pone.0189124.s011.tif]

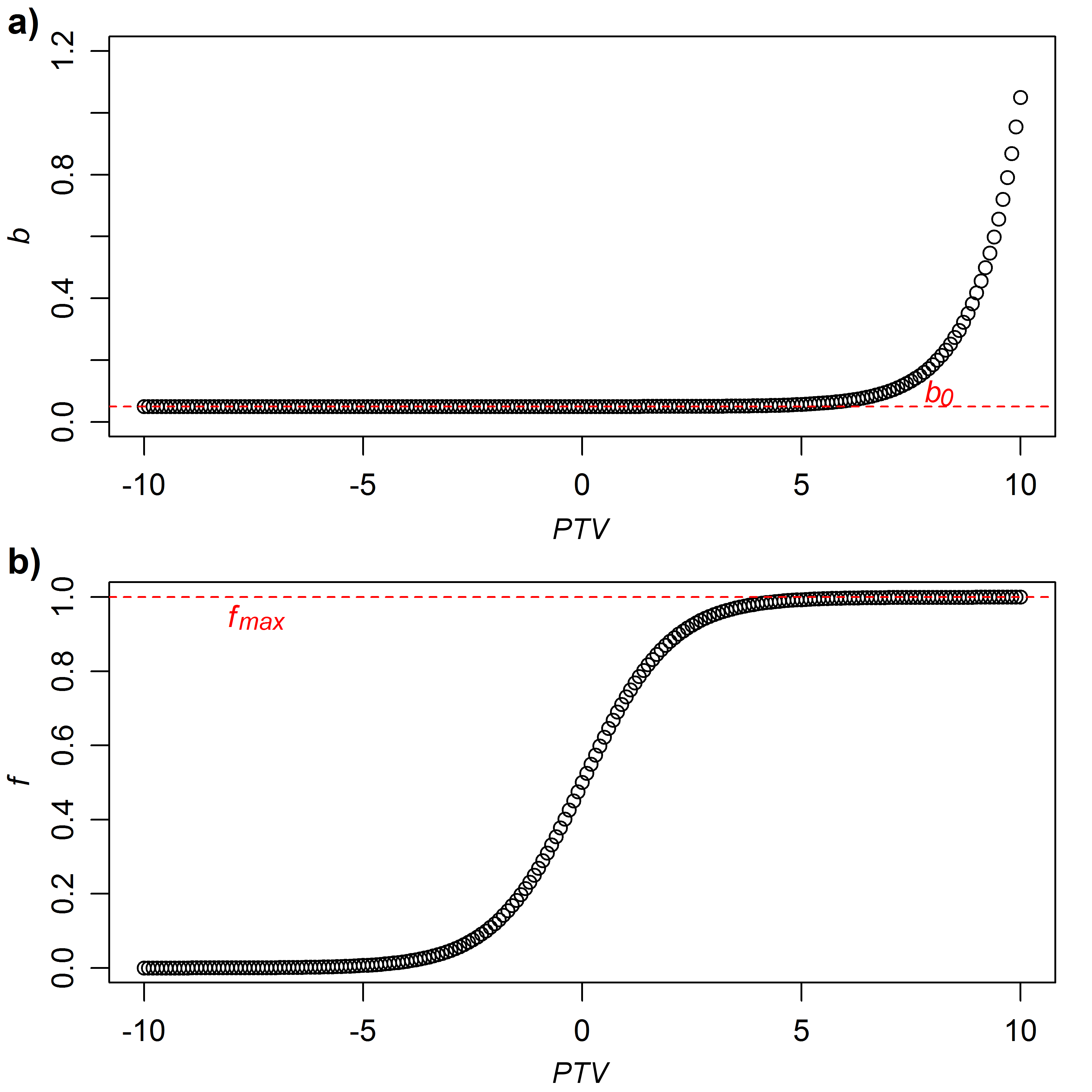

Supplement: S2 Fig — (TIF) [file pone.0189124.s012.tif]

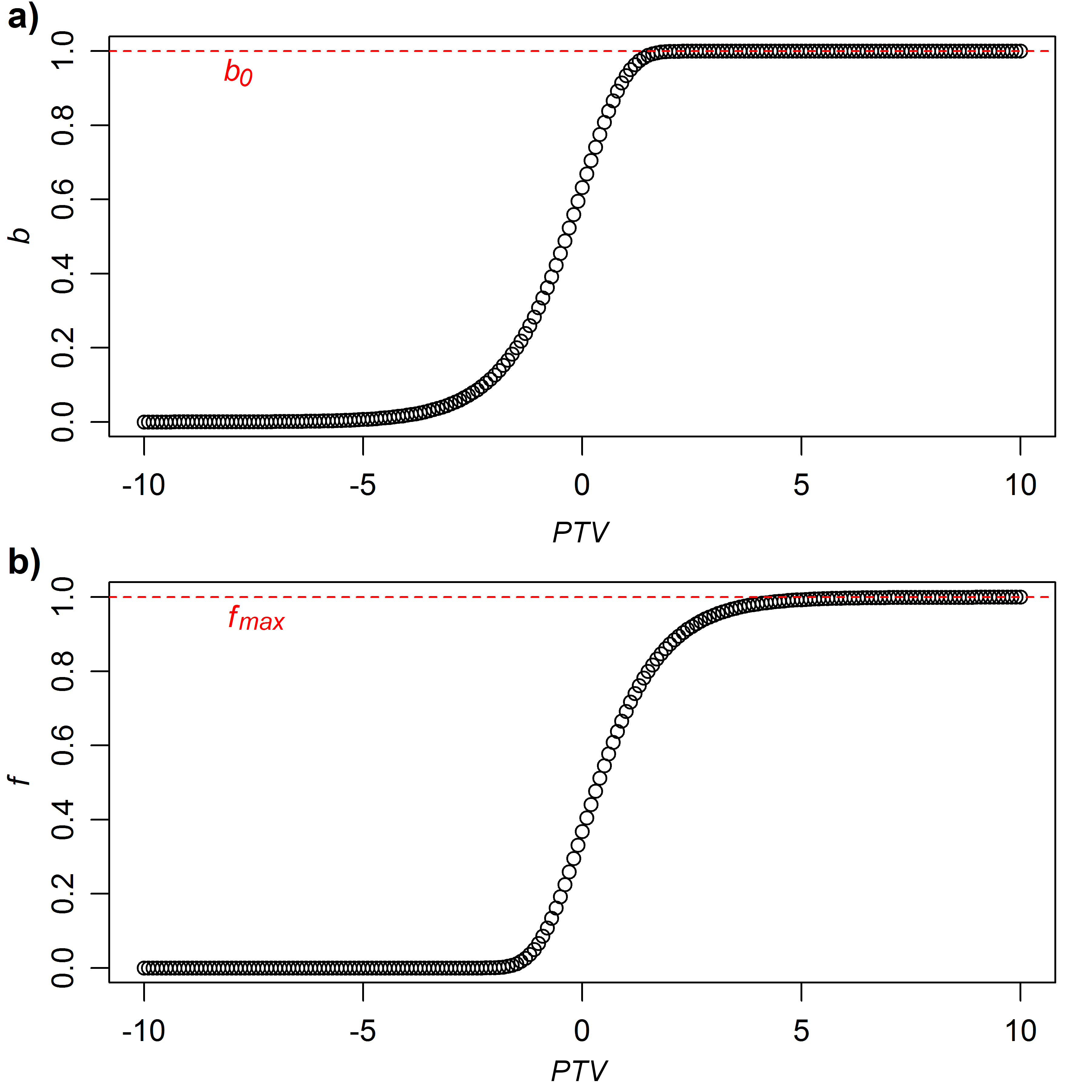

Supplement: S3 Fig — (TIF) [file pone.0189124.s013.tif]

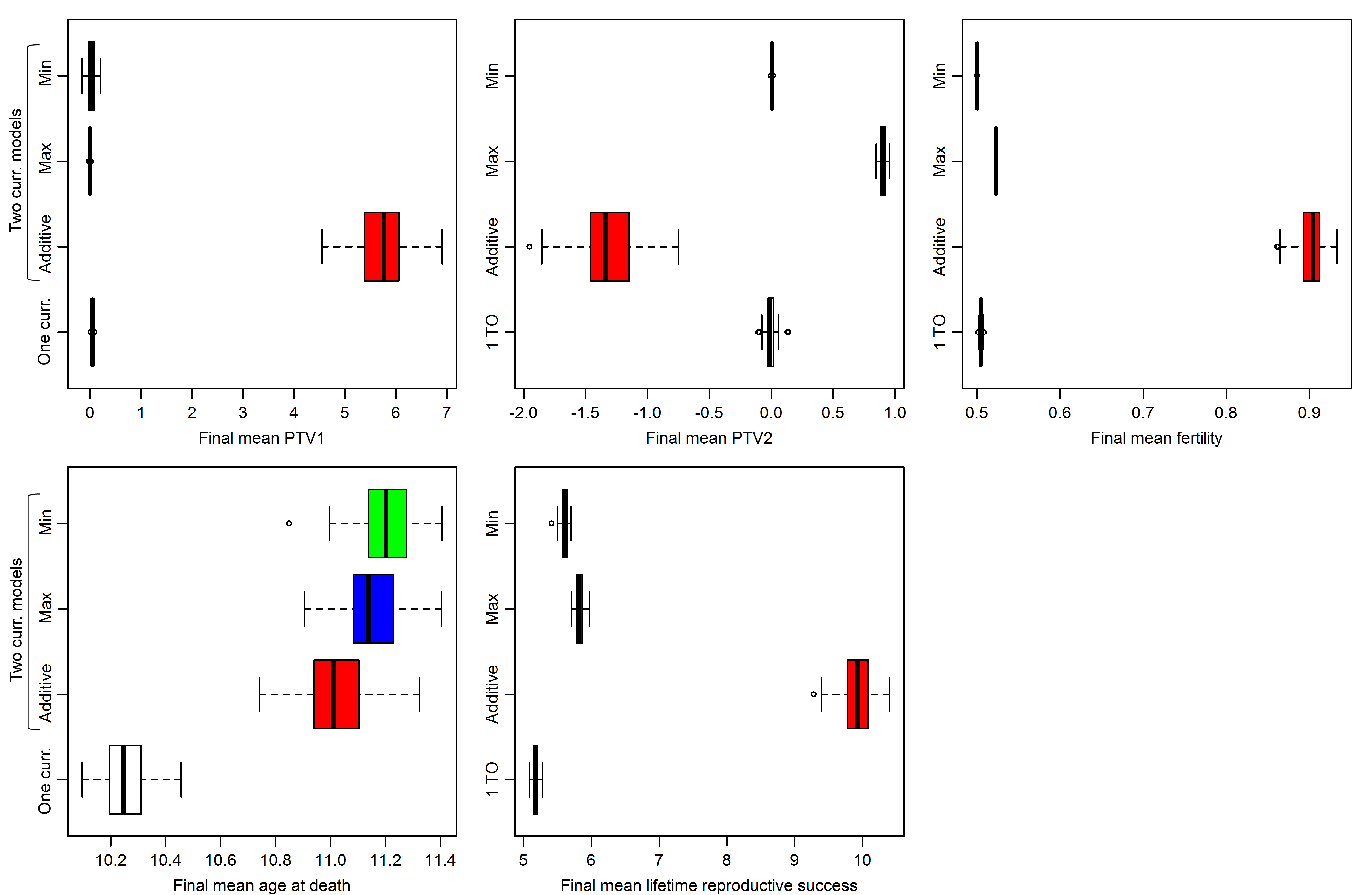

Supplement: S4 Fig — The x-axis represents the mean value of the indicated trait for the 10,000 individuals in last of the 500 generations in each simulation (boxplots are mean, interquartile range, and 1.5 times interquartile range). The four models are indicated on the y-axis. Results are largely consistent from one simulation to the next, confirming the validity of our approach to use one simulation for each parameter combination rather than the average of many. (TIF) [file pone.0189124.s014.tif]

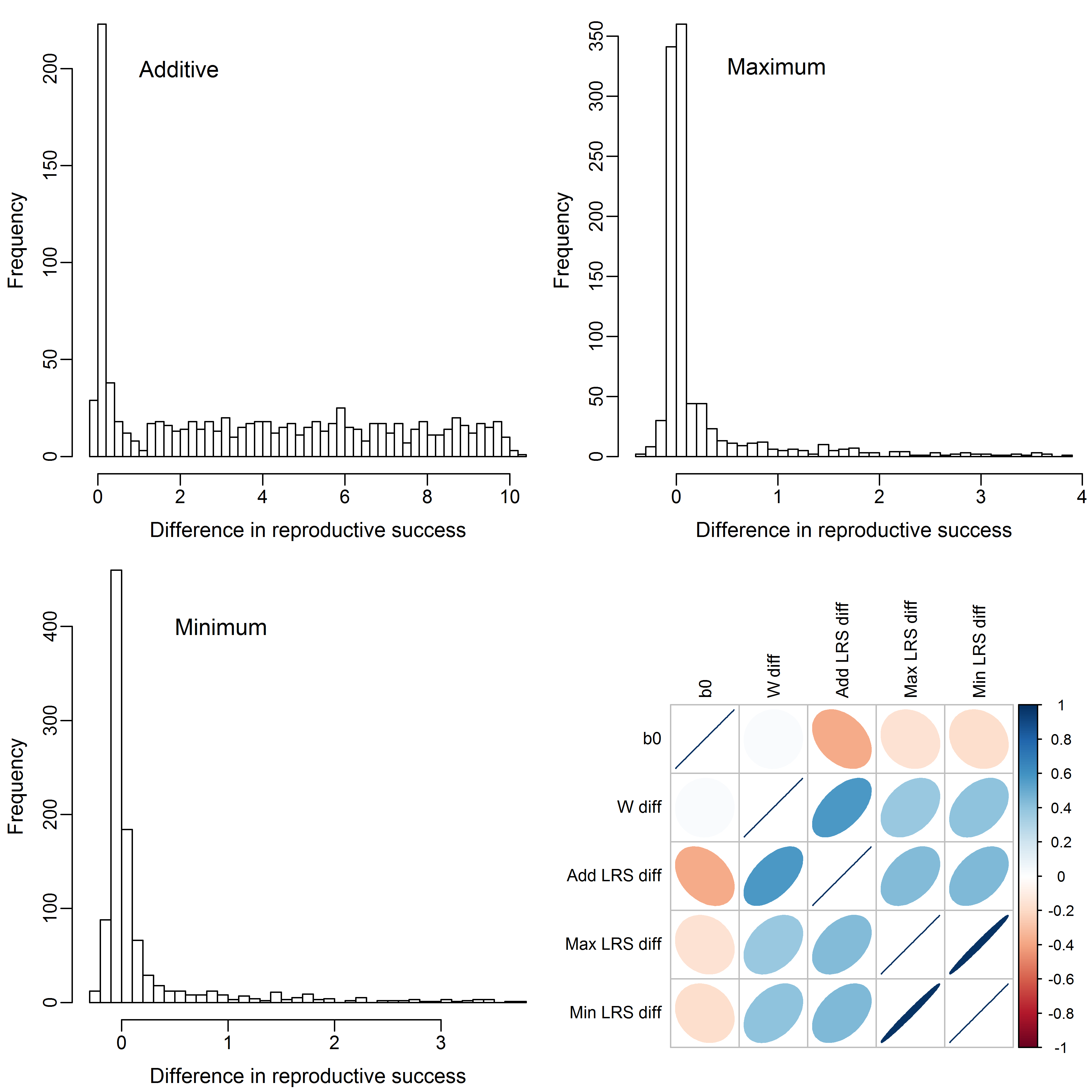

Supplement: S5 Fig — The three histograms show the respective differences between the final life time reproductive success LRS for each two-currency model and the final LRS for the one-currency model (positive indicating greater LRS in the two-currency scenario). The correlation plot represents correlations in model input parameters and results across the 1,000 runs. The shape and direction of the ellipses represent correlation strengths and directions, respectively, with correlation coefficients as indicated in the color bar (right). “W diff” indicates the difference in weights, specifically |log(W1/W2)|. This difference is strongly positively correlated with the values in the first histogram, and more weakly with the other two, implying that the additive model is increasingly effective at circumventing trade-offs as their relative effects on survival and reproduction differ more. (TIF) [file pone.0189124.s015.tif]

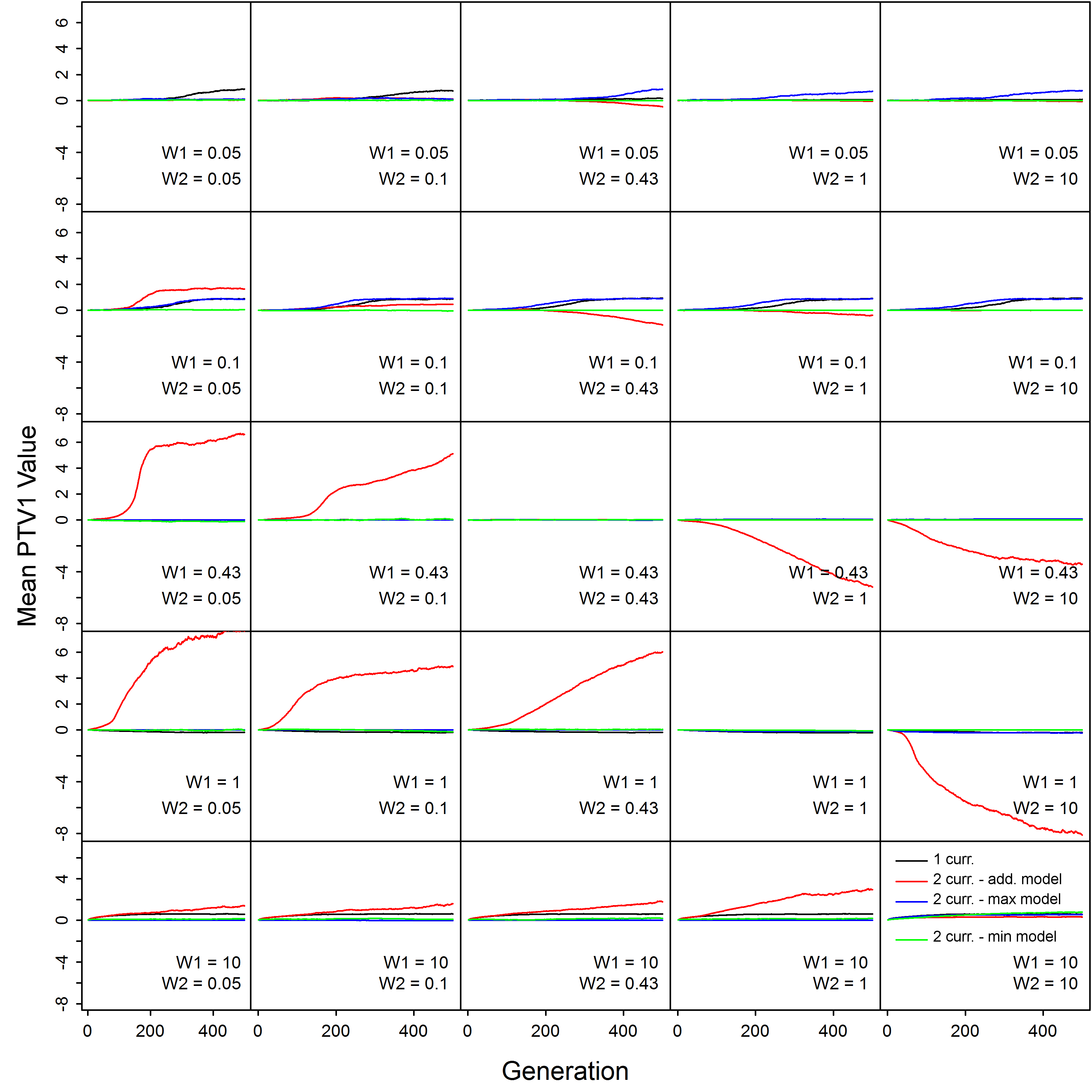

Supplement: S6 Fig — PTV1 can be considered a physiological or behavioural trait with impacts on both fertility and survival. b0 is fixed at 0.01. Effects are largest when W1 = 1, but even under other scenarios there are clear differences; see S7 Fig for finer-scale y-axes. (TIF) [file pone.0189124.s016.tif]

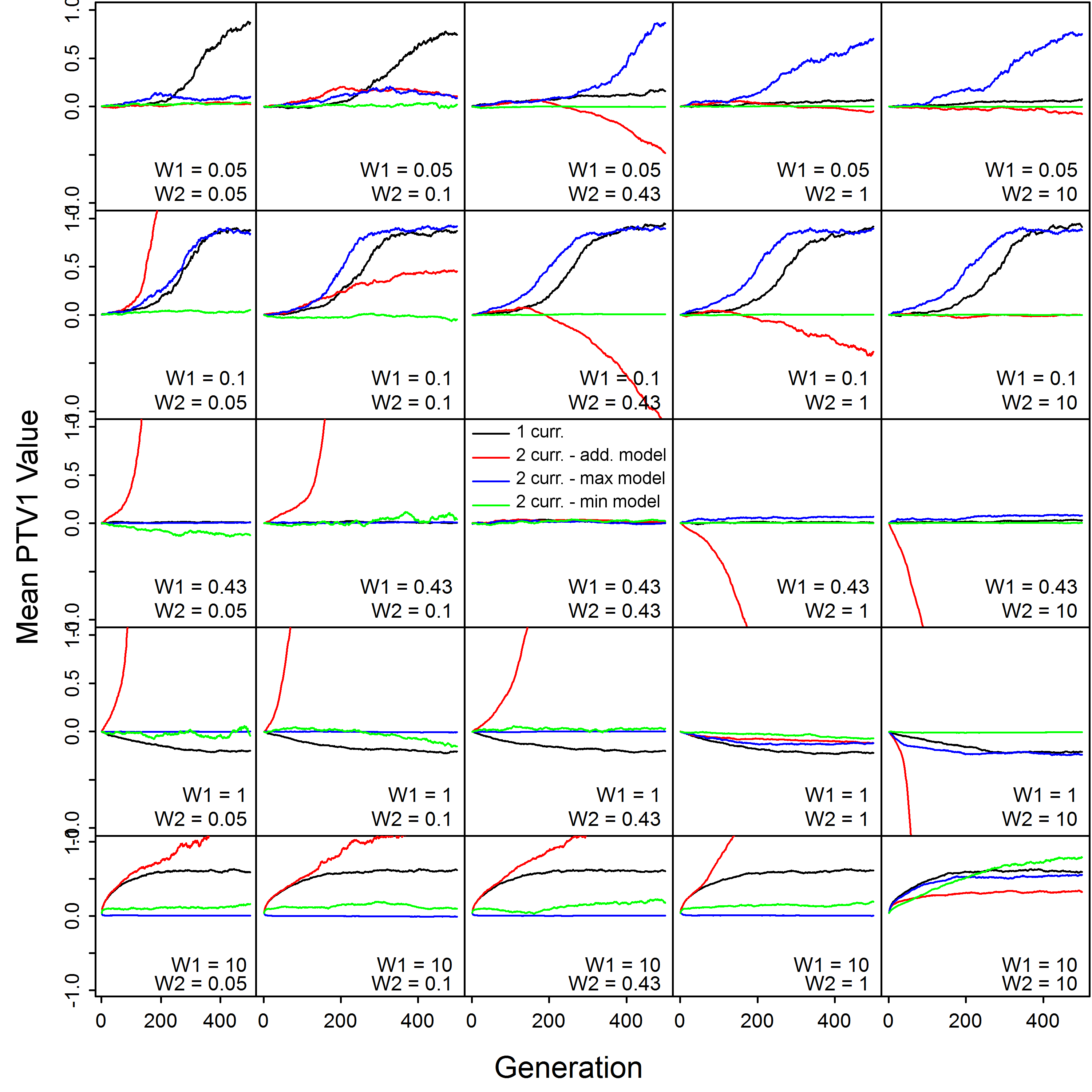

Supplement: S7 Fig — (TIF) [file pone.0189124.s017.tif]

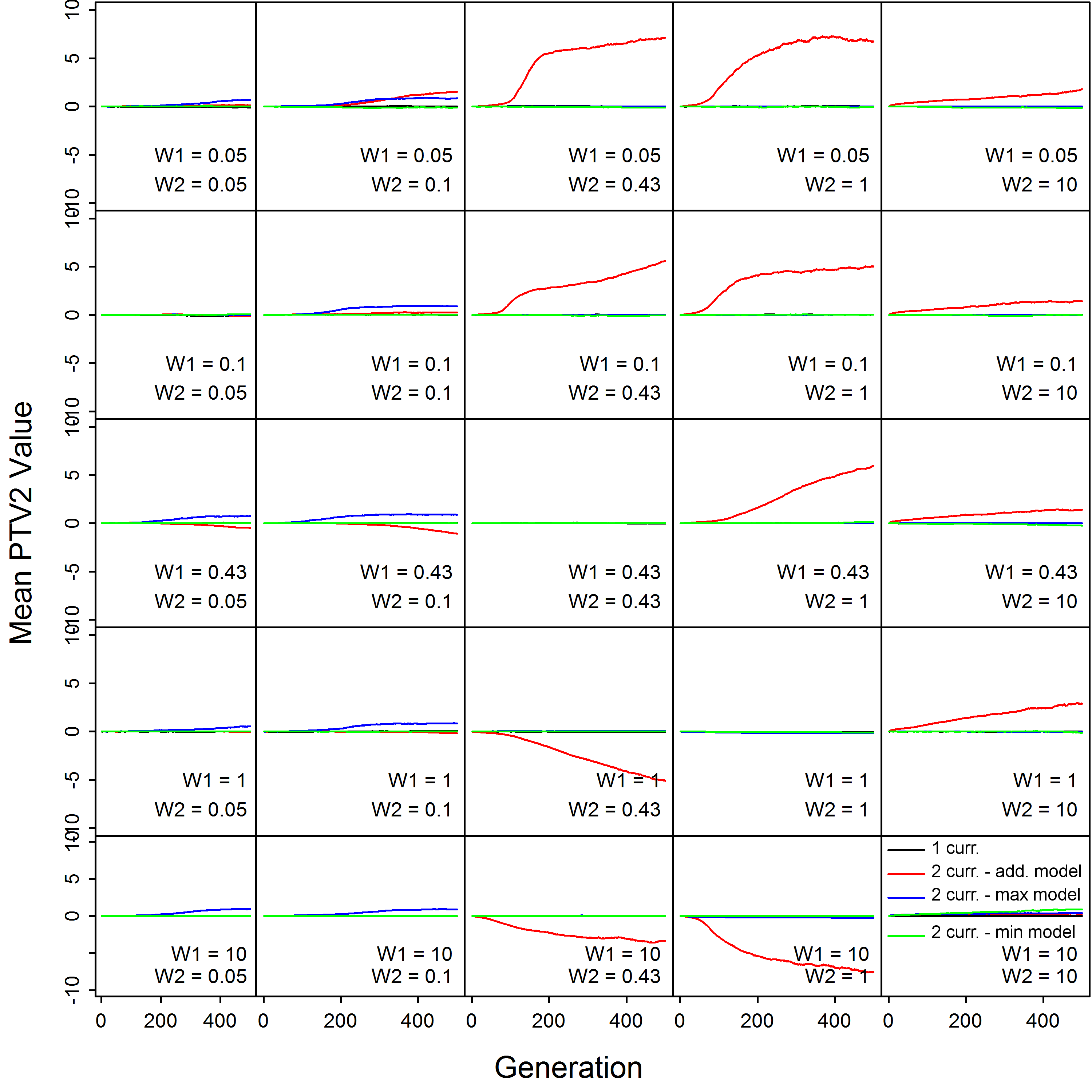

Supplement: S8 Fig — PTV2 can be considered a physiological or behavioural trait with impacts on both fertility and survival. b0 is fixed at 0.01. Effects are largest when W2 = 1, but even under other scenarios there are clear differences; see S9 Fig for finer-scale y-axes. (TIF) [file pone.0189124.s018.tif]

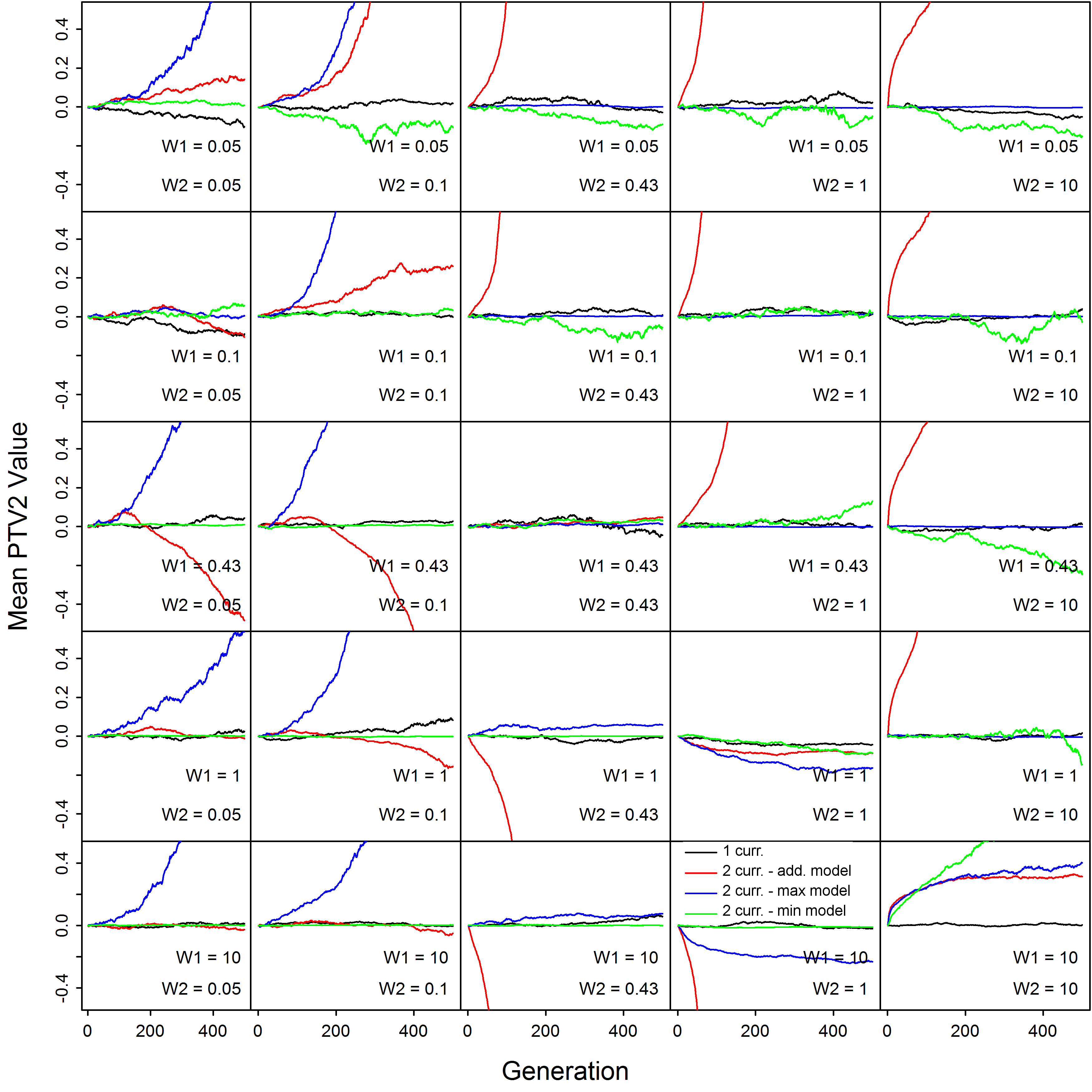

Supplement: S9 Fig — (TIF) [file pone.0189124.s019.tif]

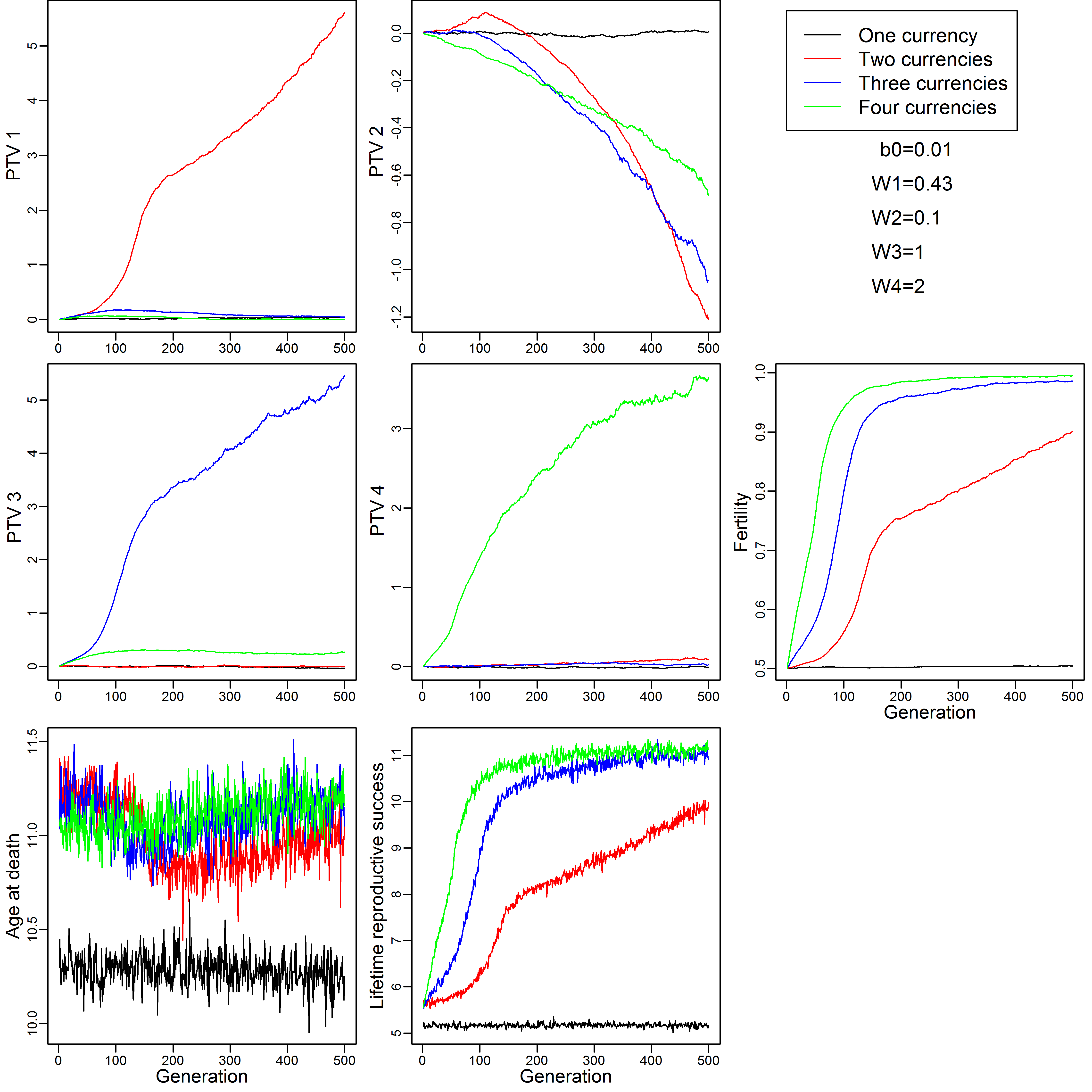

Supplement: S10 Fig — The additive model is used for two-, three-, and four-currency models, with weights as specified in the figure. Note that while each model produces a different result, the order is important: W3 and W4 are respectively more extreme than any weights in the preceding models. If the order is reversed such that successive currency weights are found within the range of preceding currency weights, no difference is seen as more currencies are added (data not shown). (TIF) [file pone.0189124.s020.tif]

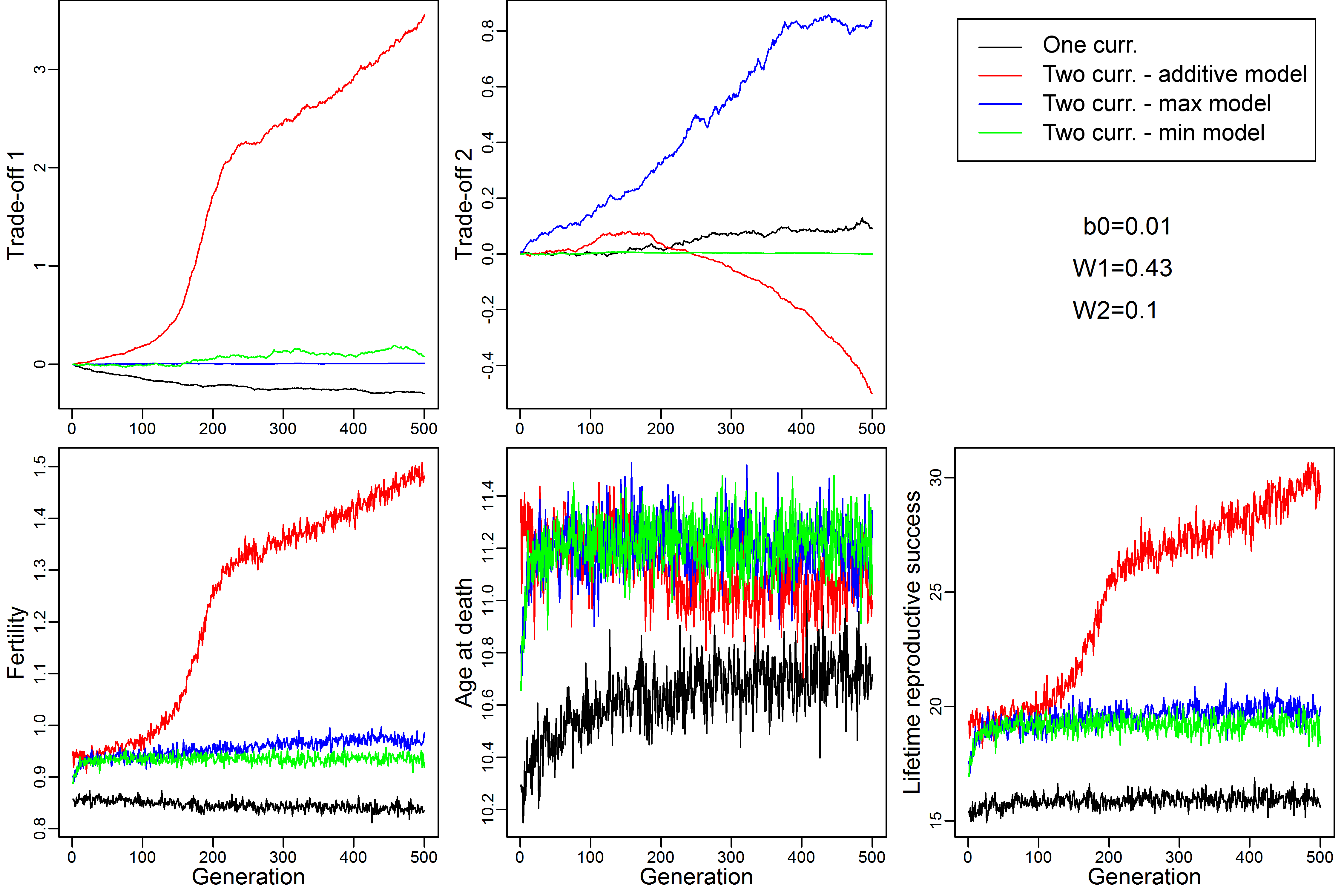

Supplement: S11 Fig — Average lifetime fertility (bottom left panel) becomes related to age at death, and consequently adds some stochasticity to this parameter relative to other models. Otherwise, results are qualitatively indistinguishable from other models. (TIF) [file pone.0189124.s021.tif]

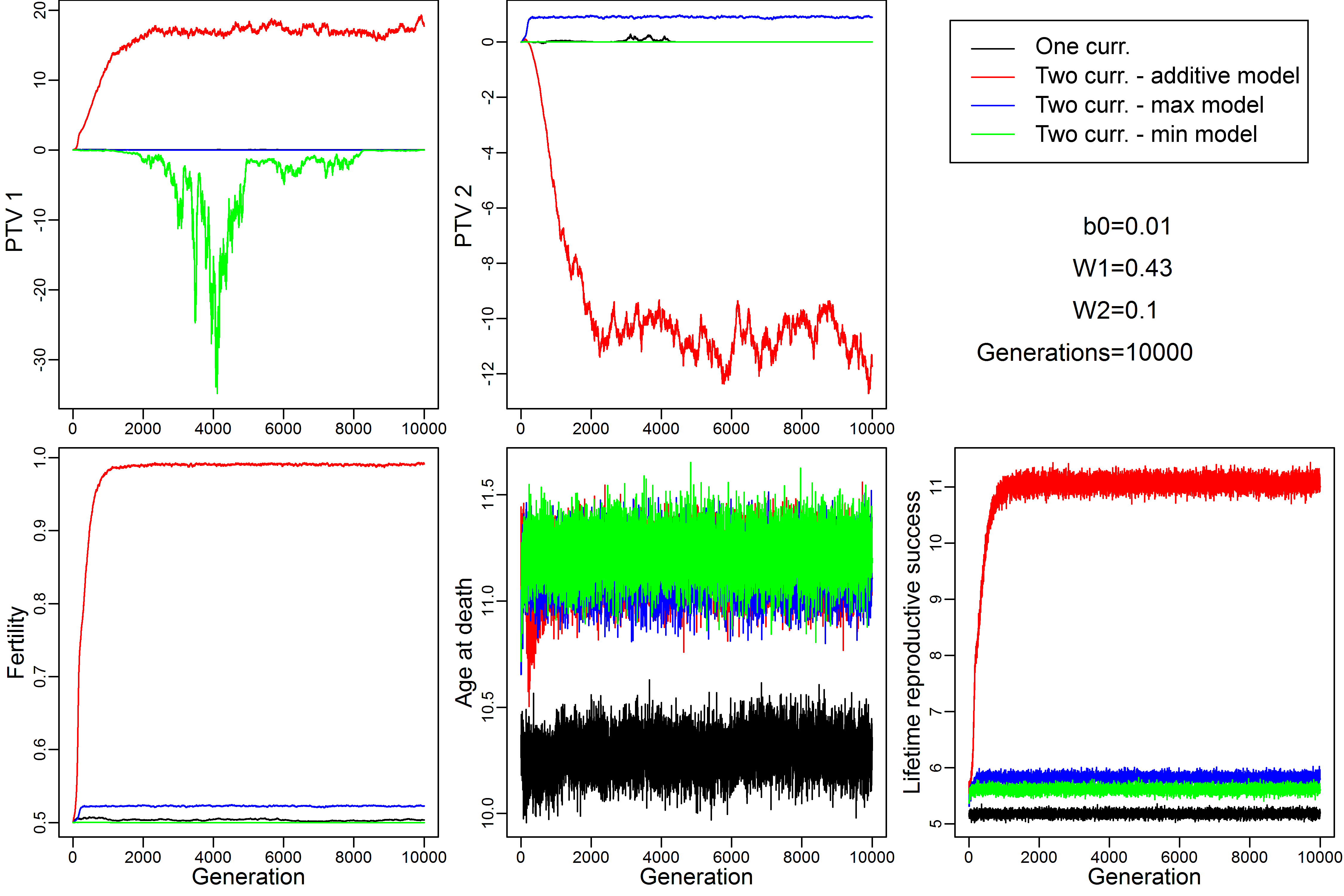

Supplement: S12 Fig — Note that convergence of life history traits is reached relatively early, suggesting that our 500-generation models are largely sufficient. Also note that physiological trait values are not stable in models with multiple currencies and can vary without much impact on life-history traits. (TIF) [file pone.0189124.s022.tif]

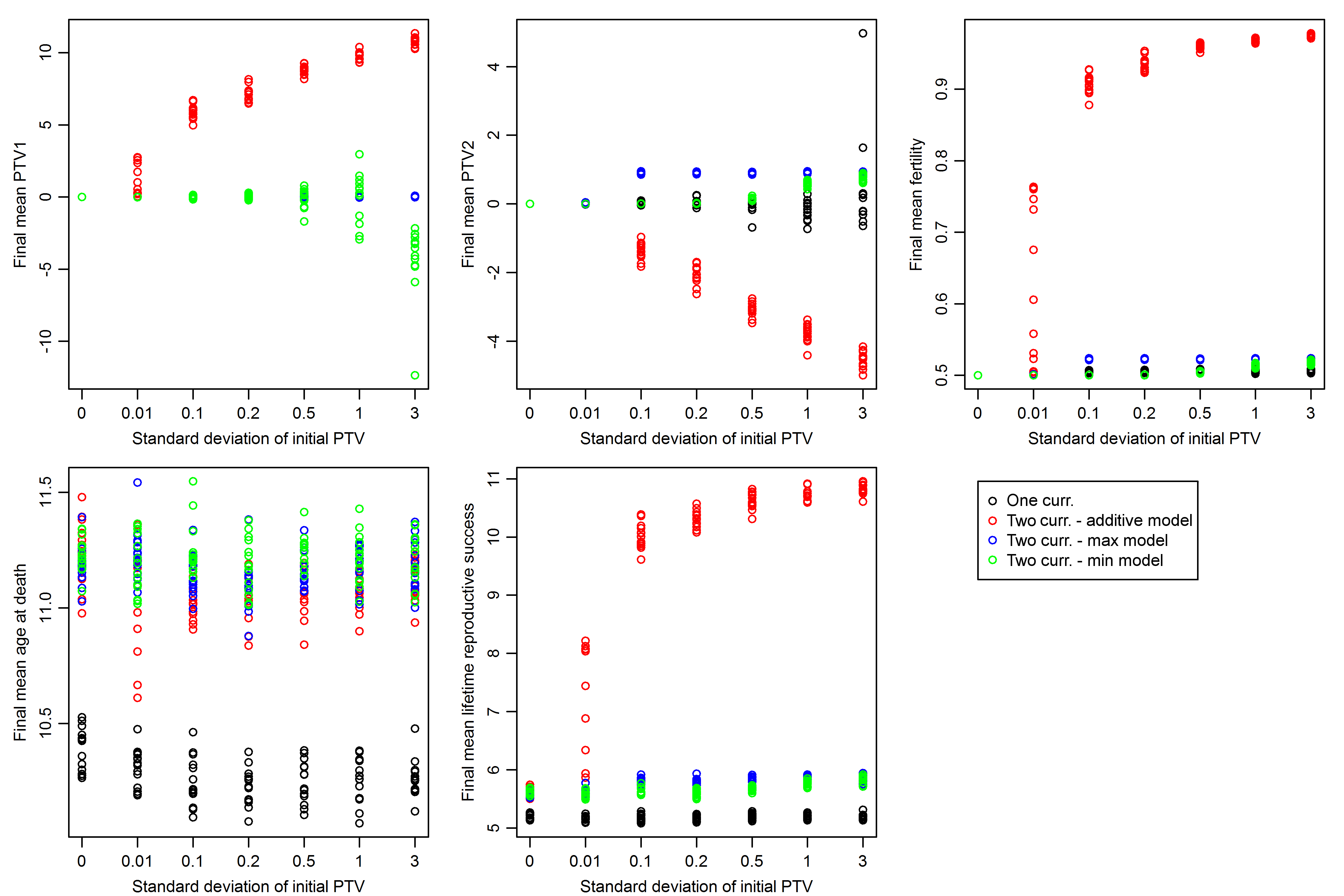

Supplement: S13 Fig — We ran 100 simulations in which this standard deviation was fixed at one of the values along the x-axis. For each of the four models in each simulation, we present the average trait value at the 500th (final) generation on the y-axis. b0 was fixed at 0.01, W1 at 0.43, and W2 at 0.1. Note that some initial variation is necessary for the model to run well, but given that heritability < 1, this variation can be minimal and model will still produce reasonable results. (TIF) [file pone.0189124.s023.tif]

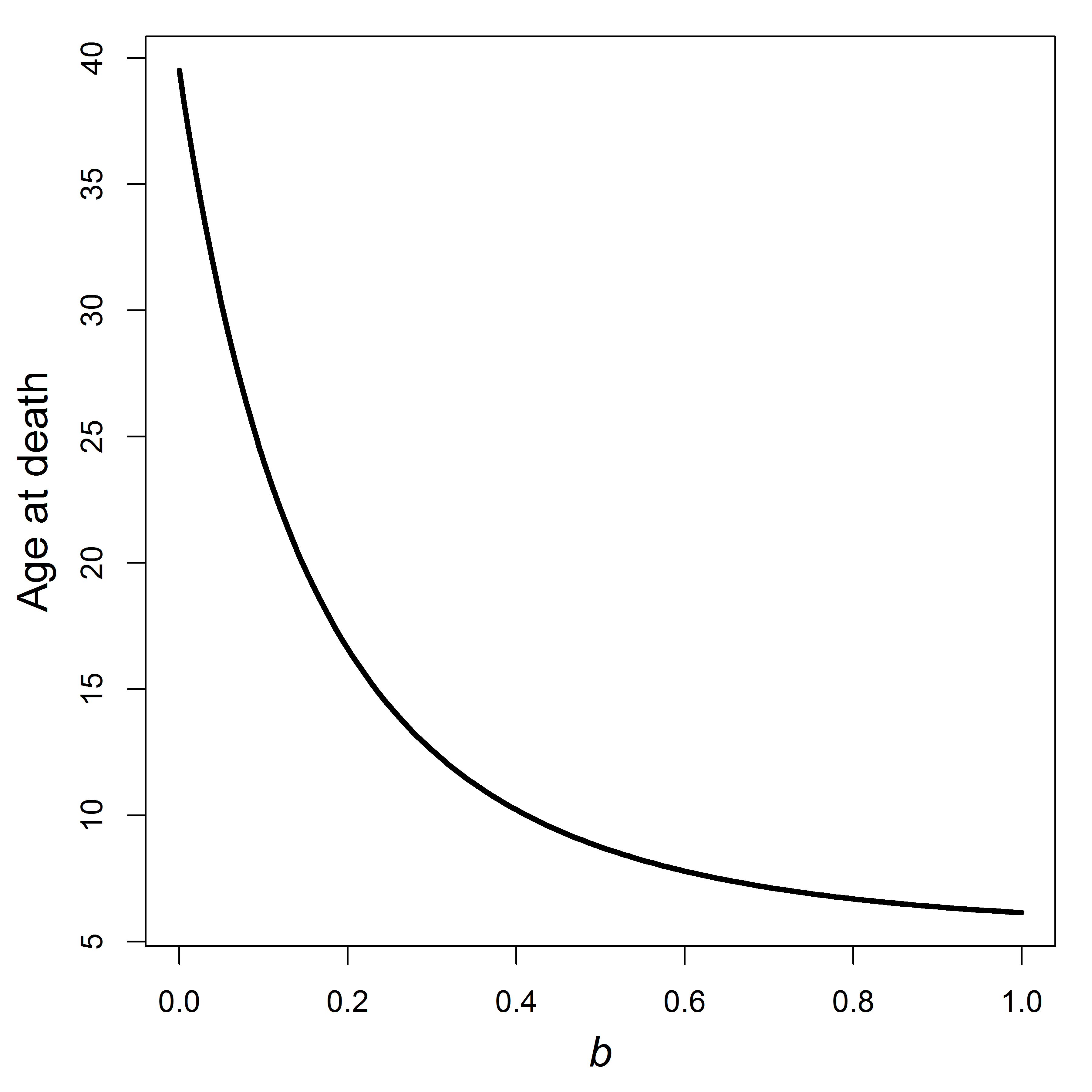

Supplement: S14 Fig — (TIF) [file pone.0189124.s024.tif]

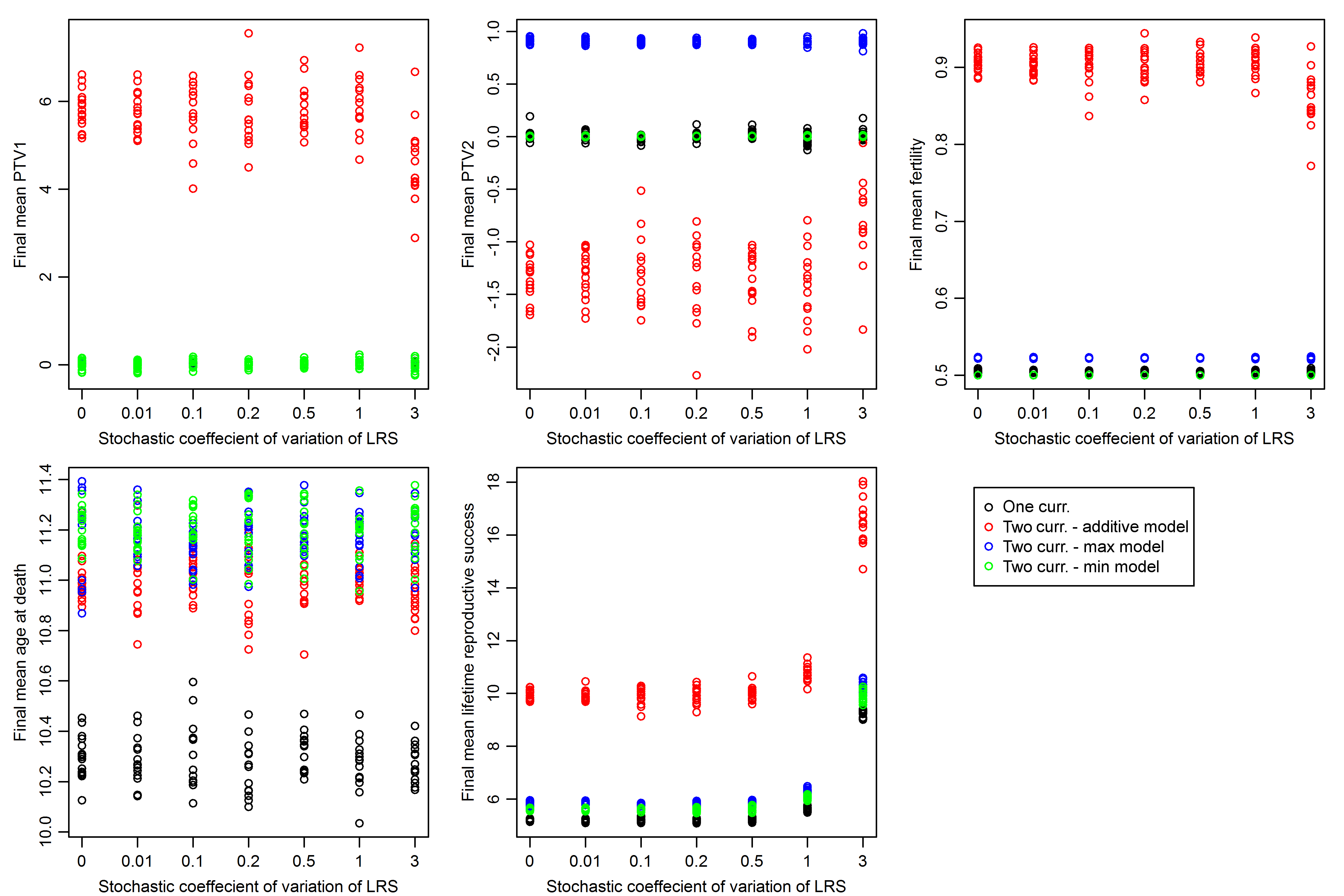

Supplement: S15 Fig — This parameter is the factor by which expected LRS is multiplied to generate the standard deviation of actual LRS: each individual’s LRS is sampled from the distribution LRS ~ N(f × Ad, f × Ad × SCV) (Eq (2)).We ran 100 simulations in which this standard deviation was fixed at one of the values along the x-axis. For each of the four models in each simulation, we present the average trait value at the 500th (final) generation on the y-axis. b0 was fixed at 0.01, W1 at 0.43, and W2 at 0.1. We use SCV = 0.1 in our simulations, but it can be seen here that the model is not sensitive to values in a wide range around this. (TIF) [file pone.0189124.s025.tif]

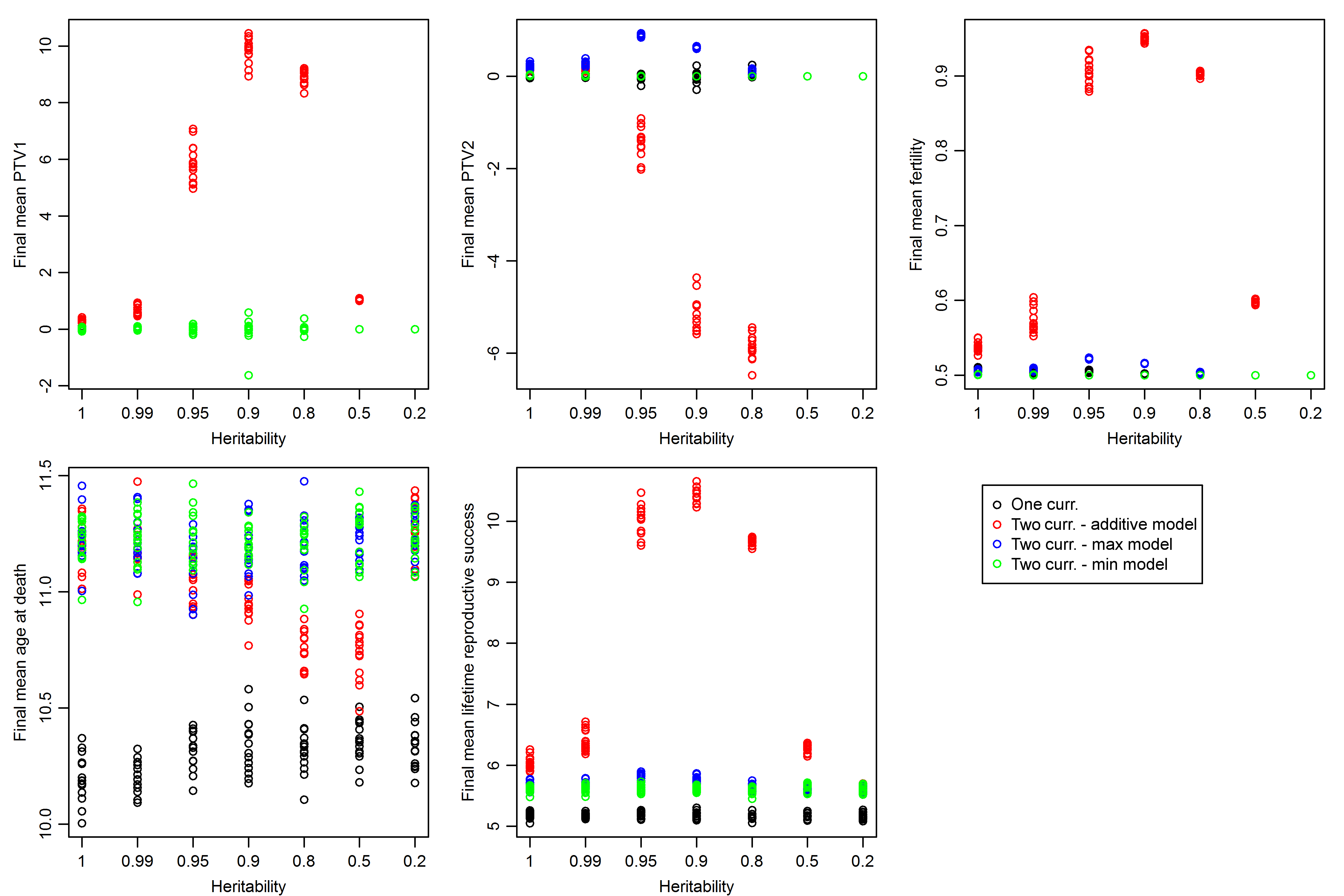

Supplement: S16 Fig — We ran 100 simulations in which heritability was fixed at one of the values along the x-axis. For each of the four models in each simulation, we present the average trait value at the 500th (final) generation on the y-axis. b0 was fixed at 0.01, W1 at 0.43, and W2 at 0.1. Note that when heritability is too high, evolution is very slow and constrained to the initial parameter range; when it is too low, stochasticity dominates. The strongest signal is thus when heritability is between 0.8 and 0.95. We use 0.95 in our models. (TIF) [file pone.0189124.s026.tif]

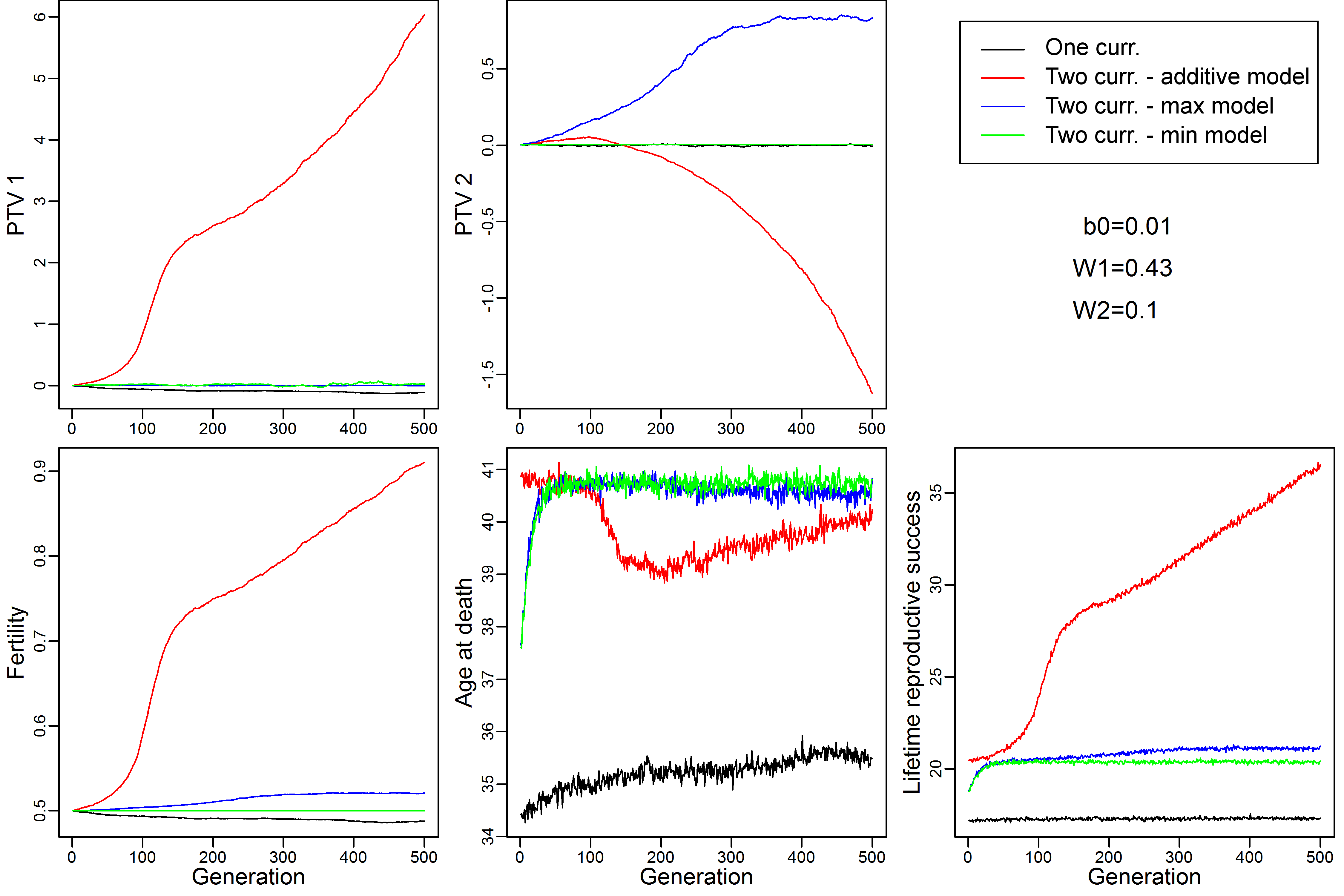

Supplement: S17 Fig — The b parameter was fixed at 0.1. Compare to Fig 2 (Gompertz function, trade-off acting on the b parameter) to see that results are not sensitive to mortality function specification. (TIF) [file pone.0189124.s027.tif]

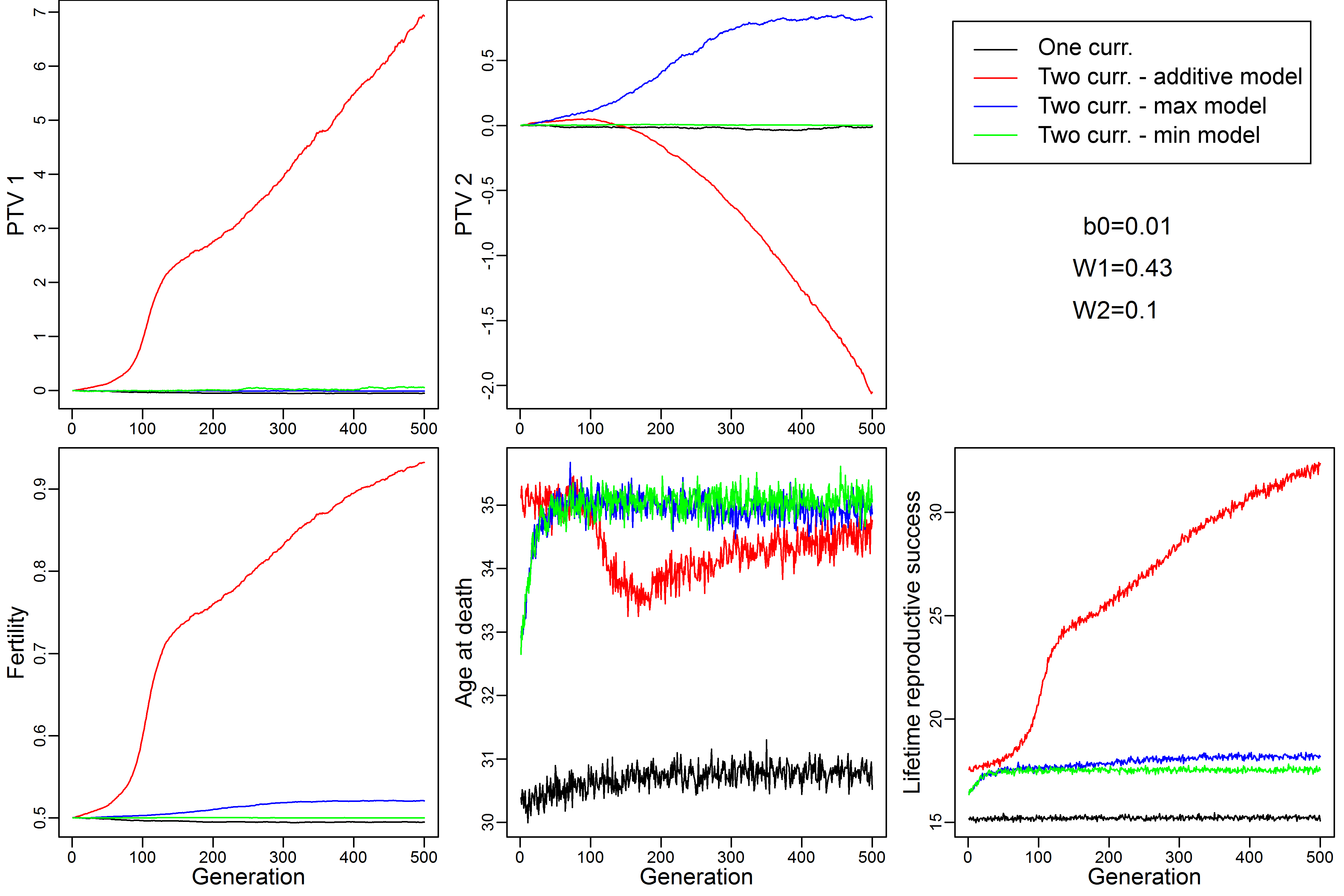

Supplement: S18 Fig — The b parameter was fixed at 0.09 and the c parameter was fixed at 0.01. Compare to Fig 2 (Gompertz function, trade-off acting on the b parameter) to see that results are not sensitive to mortality function specification. (TIF) [file pone.0189124.s028.tif]

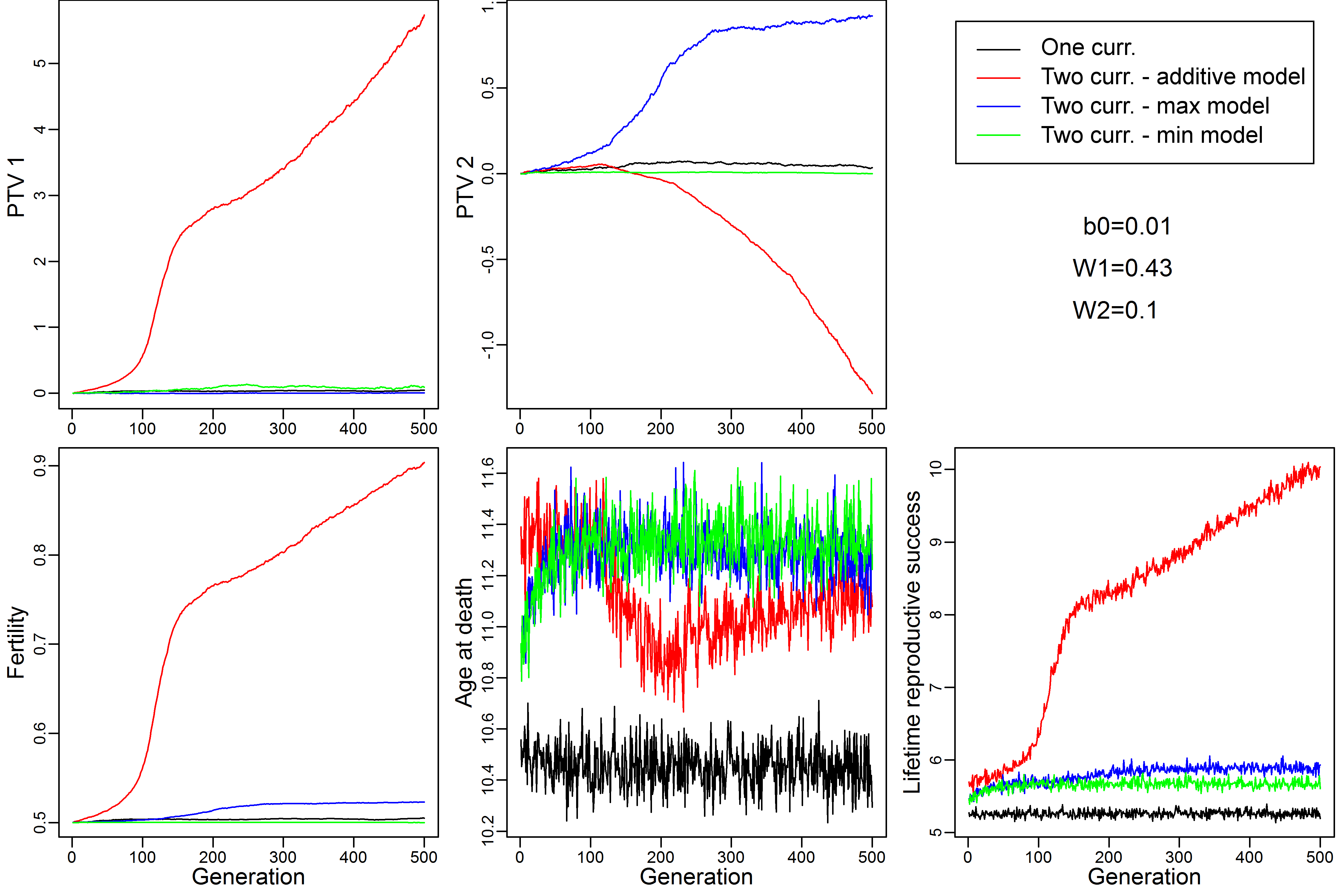

Supplement: S19 Fig — The a parameter was fixed at 0.07 and the c parameter was fixed at 0.01. Compare to Fig 2 (Gompertz function, trade-off acting on the b parameter) to see that results are not sensitive to mortality function specification. (TIF) [file pone.0189124.s029.tif]

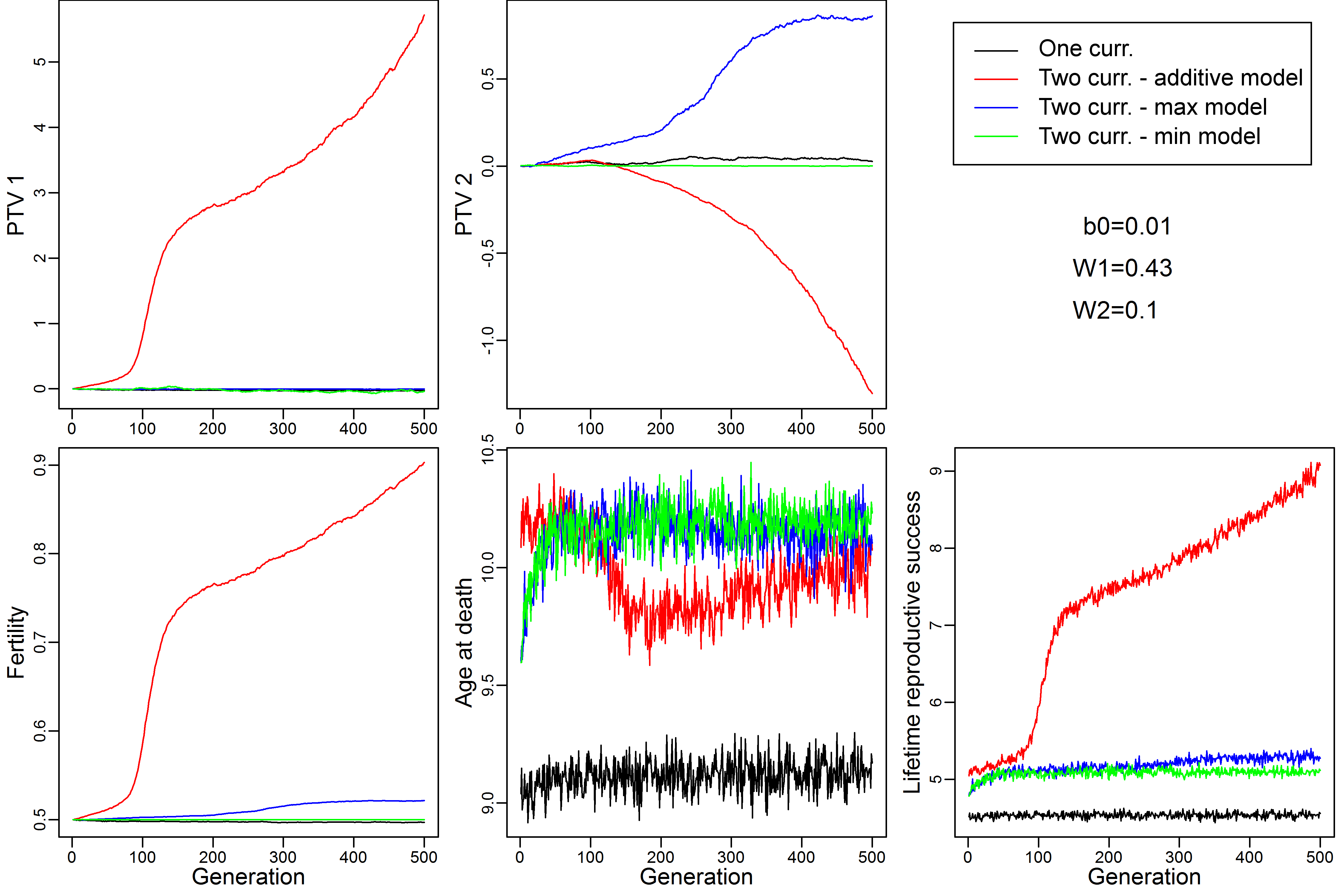

Supplement: S20 Fig — The intercept was fixed at 0.08. Compare to Fig 2 (Gompertz function, trade-off acting on the b parameter) to see that results are not sensitive to mortality function specification. (TIF) [file pone.0189124.s030.tif]

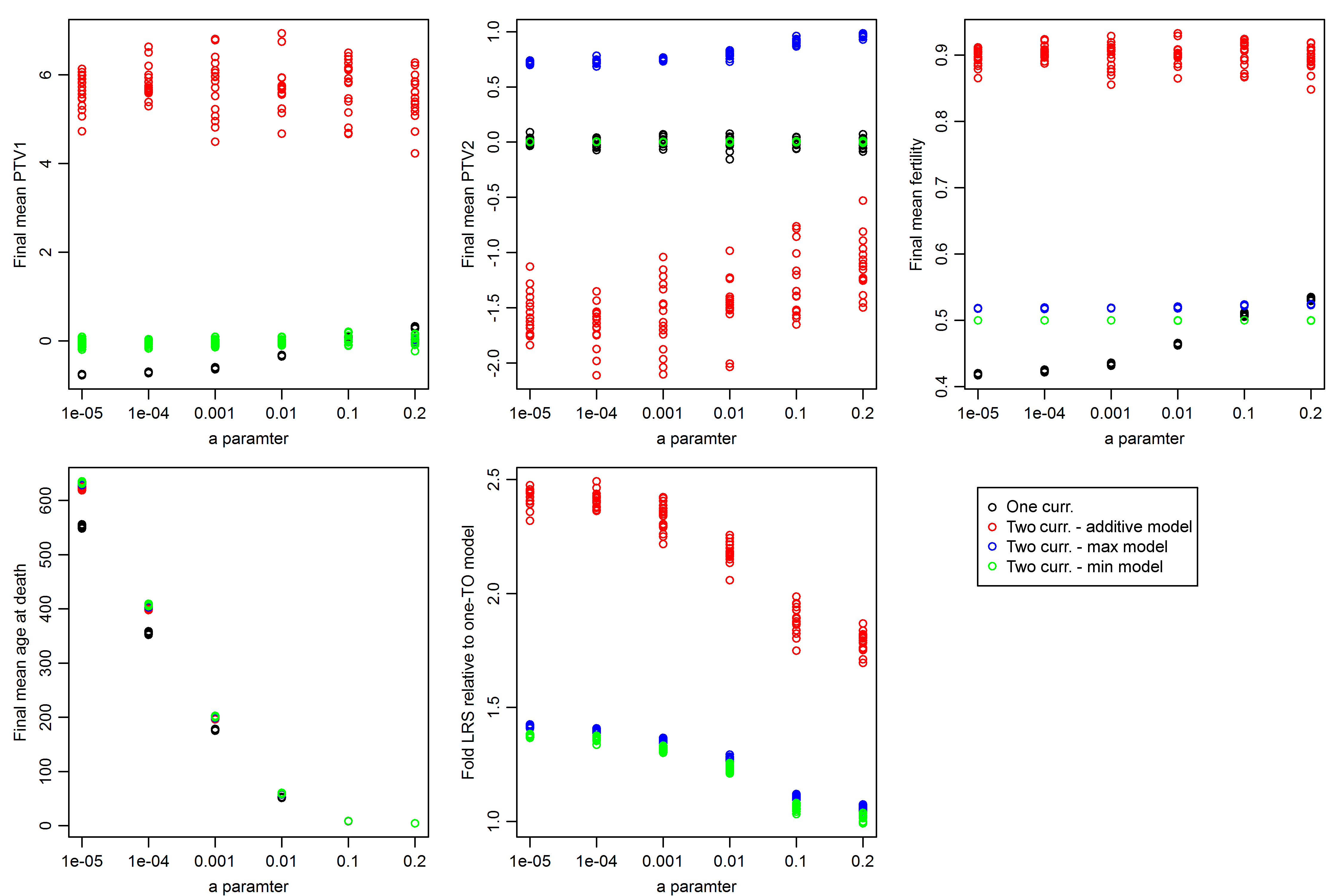

Supplement: S21 Fig — We ran 100 simulations in which a was fixed at one of the values along the x-axis. For each of the four models in each simulation, we present the average trait value at the 500th (final) generation on the y-axis. b0 was fixed at 0.01, W1 at 0.43, and W2 at 0.1. Because lifespan increases as a decreases, we scaled lifetime reproductive success (y-axis of the final panel) to the value in the single currency model. We used a = 0.08 throughout our analyses, but it can be seen here that model conclusions do not depend heavily on this parameter, and that in fact smaller values (which may be more realistic) would accentuate our findings. (TIF) [file pone.0189124.s031.tif]

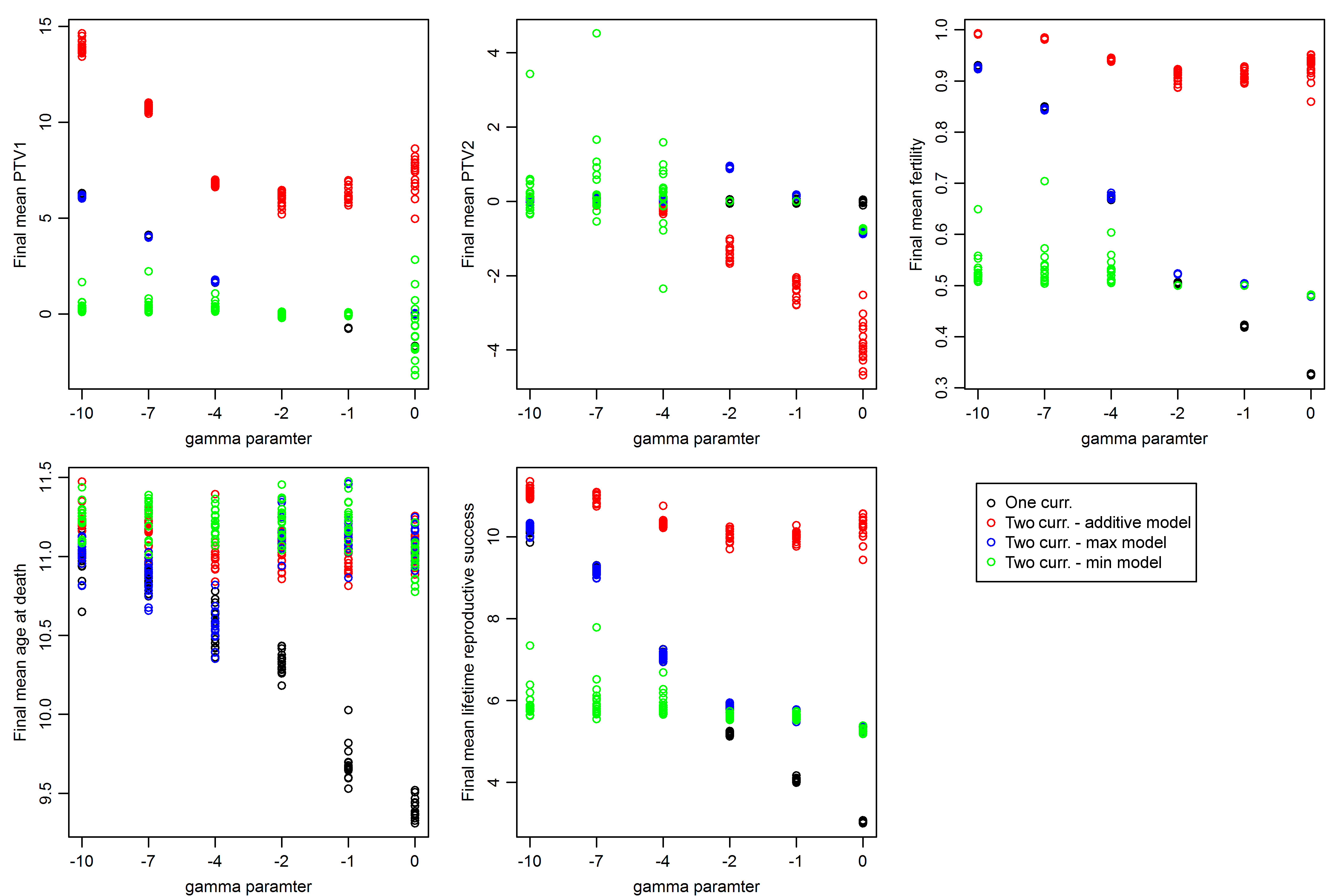

Supplement: S22 Fig — We ran 100 simulations in which γ was fixed at one of the values along the x-axis. For each of the four models in each simulation, we present the average trait value at the 500th (final) generation on the y-axis. b0 was fixed at 0.01, W1 at 0.43, and W2 at 0.1. Note that we stopped the x-axis at γ = 0 because for higher values the model often crashed: aging rate becomes so high that life expectancy goes to zero even with essentially zero fertility. As shown in S1 Fig, the key for a meaningful analysis is that γ align the key regions of variation in the fertility and aging rate functions; while extreme values are clearly problematic, this figure shows that meaningful results can be obtained at γ = -2 and for a substantial range around this value. As shown in the last panel, γ = -2 seems to be a sweet spot where the performance of the four models is most distinct. (TIF) [file pone.0189124.s032.tif]

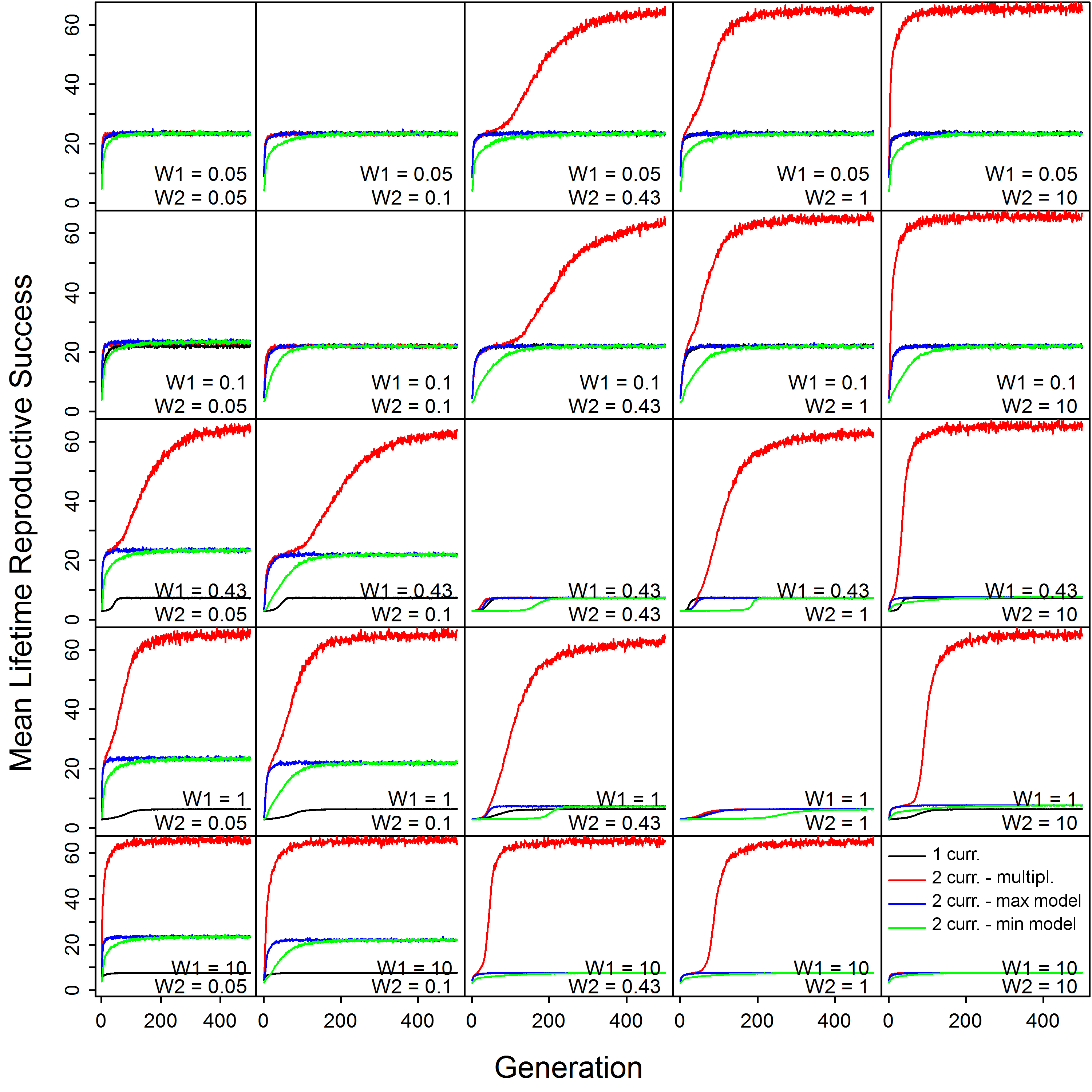

Supplement: S23 Fig — As can be seen, changing the trade-off equations has little impact on the qualitative model results in this case. (TIF) [file pone.0189124.s033.tif]
